# Discovery of RXFP2 genetic association in resistant hypertensive men and RXFP2 antagonists for the treatment of resistant hypertension

**DOI:** 10.1038/s41598-024-62804-7

**Published:** 2024-06-08

**Authors:** Shan-Shan Zhang, Lance Larrabee, Andrew H. Chang, Sapna Desai, Lisa Sloan, Xin Wang, Yixuan Wu, Nazia Parvez, Karen Amaratunga, Allison C. Hartman, Abby Whitnall, Joseph Mason, Nicholas P. Barton, Audrey Y. Chu, Jonathan M. Davitte, Adam J. Csakai, Caitlin Vestal Tibbetts, Audrey E. Tolbert, Heather O’Keefe, Jessie Polanco, Joseph Foley, Casey Kmett, Jonathan Kehler, Gabriela Kozejova, Feng Wang, Andrew P. Mayer, Patrick Koenig, Davide Foletti, Steven J. Pitts, Christine G. Schnackenberg

**Affiliations:** 1 Therapeutics Division, 23andMe, 349 Oyster Point Blvd, South San Francisco, CA 94080 USA; 2 Medicinal Science and Technology, GSK, Medicines Research Centre, Gunnels Wood Road, Stevenage, SG1 2NY UK; 3 Research, 23andMe, 223 N Mathilda Ave., Sunnyvale, CA 94086 USA; 4 Medicinal Science and Technology, GSK, 1250 S. Collegeville Rd., Collegeville, PA 19426 USA; 5 Genomic Sciences, GSK, 300 Technology Square, Cambridge, MA 02139 USA; 6 Medicinal Science and Technology, GSK, 200 Cambridgepark Drive, Cambridge, MA 02140 USA; 7 Novel Human Genetics Research Unit, GSK, 1250 S. Collegeville Rd., Collegeville, PA 19426 USA; 8 DMPK, GSK, 1250 S. Collegeville Rd, Collegeville, PA 19426 USA; 9 Bioanalysis, Immunogenicity and Biomarkers, GSK, 1250 S. Collegeville Rd., Collegeville, PA 19426 USA

**Keywords:** Genome-wide association studies, Screening, Antibody therapy, Hypertension, Mechanisms of disease

## Abstract

Hypertension remains a leading cause of cardiovascular and kidney diseases. Failure to control blood pressure with ≥ 3 medications or control requiring ≥ 4 medications is classified as resistant hypertension (rHTN) and new therapies are needed to reduce the resulting increased risk of morbidity and mortality. Here, we report genetic evidence that relaxin family peptide receptor 2 (RXFP2) is associated with rHTN in men, but not in women. This study shows that adrenal gland gene expression of RXFP2 is increased in men with hypertension and the RXFP2 natural ligand, INSL3, increases adrenal steroidogenesis and corticosteroid secretion in human adrenal cells. To address the hypothesis that RXFP2 activation is an important mechanism in rHTN, we discovered and characterized small molecule and monoclonal antibody (mAb) blockers of RXFP2. The novel chemical entities and mAbs show potent, selective inhibition of RXFP2 and reduce aldosterone and cortisol synthesis and release. The RXFP2 mAbs have suitable rat pharmacokinetic profiles to evaluate the role of RXFP2 in the development and maintenance of rHTN. Overall, we identified RXFP2 activity as a potential new mechanism in rHTN and discovered RXFP2 antagonists for the future interrogation of RXFP2 in cardiovascular and renal diseases.

## Introduction

Nearly half of adults in the United States have hypertension with prevalence higher in men up to 65 years old than women^1^. Individuals with hypertension (> 130/80 mmHg) despite taking three antihypertensive drugs or who achieve goal levels on ≥ 4 drugs are defined as having resistant hypertension (rHTN) and are at greater risk for cardiovascular disease (CVD), chronic kidney disease (CKD), and death^2,3^. The estimated prevalence of rHTN among hypertension in US adults is 12–18%^2^. CVD is the leading cause of death in the US with approximately 13% attributed to hypertension^1^. CKD affects 14% of US adults^4^ and 1 in 9 adults worldwide with a mortality rate 1.39 times greater among men than women^3^. Hypertension is one of the leading causes of CKD and responsible for 43.2% of CKD disability-adjusted life years worldwide. In some countries, hypertension is the number one cause of CKD^3^. Lowering blood pressure improves cardiovascular and renal outcomes and slows the progression to end stage kidney disease^5^; however, hypertension and the resulting CVD and CKD remain large healthcare burdens.

Blood pressure is a complex pathophysiological condition that is influenced by genetics such as male sex^2,6^, lifestyle, pharmacological agents^2^, psychosocial and environmental factors^7^, and other health conditions^2^. Several mechanisms that include the endocrine, renal, neural, cardiac, metabolic, and vascular systems are important in the regulation of blood pressure^8^. The adrenal gland secretes mineralocorticoids (e.g. aldosterone) and glucocorticoids (e.g. cortisol) which are critically important hormones in electrolyte and fluid homeostasis and if left unchecked can increase blood pressure. Abnormally high corticosteroid levels cause electrolyte and fluid imbalance resulting in hypertension and diseases such as Cushing’s Syndrome and primary aldosteronism. Mineralocorticoid receptor antagonists are current treatments for hypertension, with particular use in rHTN^2,8^. Despite several classes of drugs which target multiple mechanisms, there remains a high unmet medical need for a new class of antihypertensive therapy. In addition, the pathophysiology of rHTN remains unclear and genetic technologies provide a growing opportunity to uncover new genetic contributions and potential new therapies. Furthermore, mechanisms responsible for the sex difference in hypertension and rHTN have not been fully elucidated.

The relaxin family peptide receptor 2 (RXFP2) is a class A G-protein coupled receptor (GPCR) that is expressed in testis, gubernaculum, ovary, kidney, brain, muscle, bone, thyroid, and adrenal gland^9–17^. The protein is highly conserved across human and rodent species and has unusual structural and functional characteristics^18,19^. In addition to seven transmembrane alpha-helices, RXFP2 also has a large extracellular domain (ECD) with 10 leucine-rich repeats (LRRs) which is the primary ligand binding site, a linker region that is required for activation, and a single low-density lipoprotein receptor class-A (LDLa) module at the N-terminus which is essential to receptor signaling. Two extracellular loops are also likely required for signaling^18,20–23^. Insulin-like peptide 3 (INSL3) and relaxin (RLN2) are two related high affinity ligands of RXFP2, but INSL3 is the only known physiological ligand of RXFP2 ^18^. INSL3 is expressed in testis and ovary, circulates at higher concentrations in men than women, and increases intracellular cAMP generation upon binding RXFP2^9,15,21,23–26^. Although the INSL3/RXFP2 system has been studied primarily for its specialized roles in testis descent, spermatozoa maintenance, and steroidogenesis in the ovary, it may have other roles in the adrenal gland, central nervous system, kidney, bone growth, and cancer metastasis^9,15,17,19,24,26,27^. Current pharmacological tools to investigate the physiological and pathophysiological roles of RXFP2 are limited mostly to protein and small molecule RXFP2 agonists^28^. Although some INSL3 peptides have been demonstrated to block RXFP2, their in vivo use is restricted by a short half-life reflecting their rapid proteolysis and renal clearance^29–33^.

Herein we conduct genome-wide association studies to identify new potential causes of resistant hypertension and report that RXFP2 is associated with rHTN in men. A RXFP2 expression quantitative trait locus (eQTL) in the adrenal gland indicates higher expression of RXFP2 is associated with increased risk of rHTN. We show that RXFP2 gene expression is increased in adrenal glands from hypertensive men. Because of the importance of adrenal corticosteroids in the regulation of blood pressure, we elucidate the pathophysiological role of RXFP2 in the adrenal gland using newly discovered small molecule and mAb blockers of RXFP2. We determine that the neutralizing RXFP2 mAbs are suitable for use in rodents to further explore the role of RXFP2 in rHTN and potentially other diseases.

## Results

### RXFP2 genome-wide association study (GWAS)

**Figure 1:**
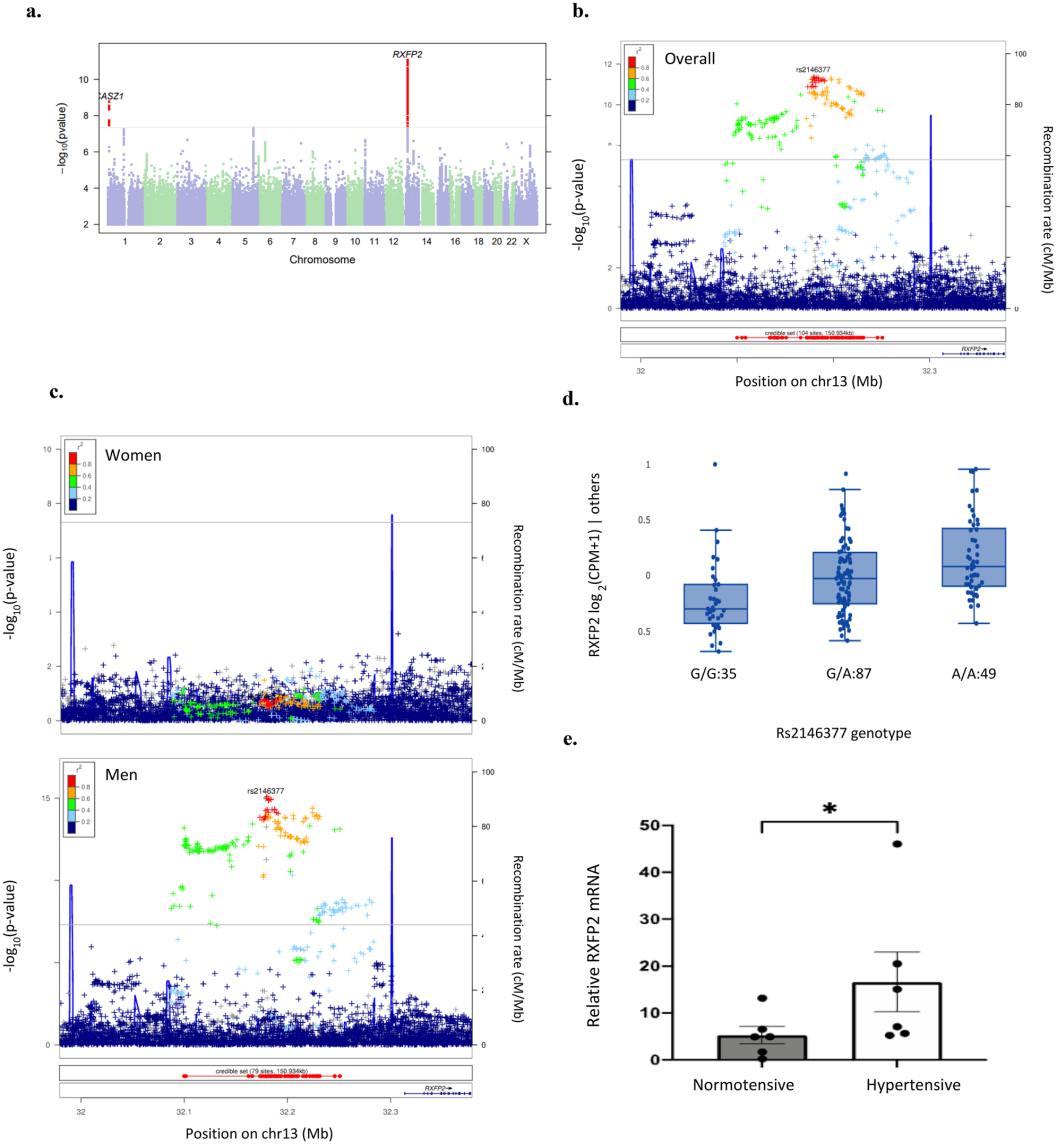
RXFP2 is associated with resistant hypertension in men. (**a**) Manhattan plot of GWAS on resistant hypertension versus non-resistant hypertension in the 23andMe cohort. Locuszoom plots of the association upstream of RXFP2 in both men and women (**b**) and in women and men separately (**c**). The lead SNP (rs2146377) is highlighted. (**d**) RXFP2 allelic status is associated with RXFP2 gene expression changes in the adrenal gland (GTEx v8, www.gtexportal.org/home/snp/rs2146377, *p* = 1.4e-14). (**e**) Endogenous RXFP2 mRNA expression relative to beta-actin in adrenal gland of hypertensive men (n = 6) is increased compared to normotensive men (n = 6, *p* = 0.041, Mann–Whitney U test).

We collected the number of anti-hypertensive medications taken in a 23andMe survey and conducted a GWAS contrasting individuals with rHTN (> 3 medications, n = 14,954) and non-rHTN (≤ 3 medications, n = 502,351). Genome-wide significant associations were detected at loci near CASZ1 and RXFP2 (Fig. 1a). CASZ1 was previously associated with cardiovascular traits, including blood pressure, stroke, and rHTN^34,35^. Both loci were reported for primary aldosteronism^17,36^. The novel rHTN signal lies upstream of RXFP2 (rs2146377, MAF = 0.43, OR = 0.92 [0.90, 0.94], λ = 1.030, *p* = 8.6e-12) and RXFP2 is the nearest gene. A sex-stratified analysis revealed that this association is observed only in men (*p* = 1.1e-15; OR = 0.88 [0.85, 0.9]) but not women (*p* = 0.25, OR = 0.98 [0.94, 1.02]) (Fig. 1b–d). To account for the potential false discovery, we replicated the association found in the 23andMe cohort using the UK Biobank as an independent cohort. We found that the association between rs2146377 at the RXFP2 locus is replicated and shows consistently higher effect size in men than women (Men: OR 1.20, *p* = 4.3e-7; Women: OR 1:04, *p* = 0.34) (Supplementary Fig. S1).

Using data from the GTEx portal (www.gtexportal.org/home/), we determined that rs2146377 is an expression quantitative trait locus (eQTL) for RXFP2 associated with higher expression in the adrenal gland (*p* value = 1.4e-14) (Fig. 1e), indicating that rHTN disease risk is associated with adrenal RXFP2 mRNA. rs2146377 is an eQTL for RXFP2 in the thyroid gland (*p* value = 7.5e-6) though not the SNP with the smallest *p* value in that tissue (rs9603300, *p* value = 3.9e-7), and is not an eQTL for any other gene. Colocalization analysis between the rHTN GWAS and RXFP2 eQTL in the adrenal gland shows strong evidence of shared genetic factors (PP4 [posterior probability for a shared causal variant] = 0.93)^37^. The same colocalization analysis with the thyroid gland eQTL does not show significant evidence of shared genetics (PP4 = 0.60; data not shown). The directionality derived from colocalization indicates that genetic factors associated with higher risk of rHTN are also associated with higher adrenal RXFP2 expression, which is consistent with the known biological role of the adrenal gland in hypertension. We hypothesize that antagonizing adrenal RXFP2 may have a therapeutic benefit in rHTN.

### RXFP2 phenome-wide association study (PheWAS)

To identify diseases or traits that may be associated with rHTN and RXFP2 expression, we performed a PheWAS for rs2146377 in the 23andMe cohort, across publicly available UK Biobank PheWAS results [www.nealelab.is/blog/2017/9/11/details-and-considerations-of-the-uk-biobank-gwas], and publicly available FinnGen PheWAS results^38^. Our analysis revealed significant associations of rs2146377 with hyperaldosteronism and other hypertension-related conditions (Supplementary Figs. S2–S4, *p* < 1e-5).

### RXFP2 expression in adrenal gland

Adrenal gland from hypertensive and normotensive Caucasian men (n = 6 each) were studied. Samples from individuals with evidence of hypertension, heart disease, or cardiovascular disease were excluded from the normotensive cohort. Individuals with cancer were excluded from both the hypertensive and normotensive cohorts (Supplementary Table S1). Relative gene expression analysis conducted via RT-qPCR revealed that adrenal RXFP2 mRNA is more abundant by 3.16-fold in hypertensive compared to normotensive men (*p* = 0.041) (Fig. 1e). Regional bias of tissue collection was ruled out by using genetic markers of adrenal cortex (NOV) and adrenal medulla (CHGB)^39^. No significant difference in the gene expression of NOV or CHGB between adrenal tissue samples from normotensive and hypertensive samples was detected (Supplementary Fig. S5).

### Small molecule RXFP2 antagonist discovery

A high throughput screen (HTS) assay was developed using HEK293 cells transduced with RXFP2 via baculovirus-mediated gene transfer (BacMam)^40^. RXFP2 antagonists were identified using a primary, single-shot antagonist screen (10 µM) of the GSK compound library. Putative ‘hits’ were confirmed by 11-point dose–response curve with 1:3 dilutions from 100 µM. The primary assay was performed using homogenous time resolved fluorescence (HTRF) to detect compound inhibition of INSL3 EC_80_-induced cAMP in HEK293 expressing human RXFP2 (hRXFP2; INSL3 EC_50_ = 0.048 nM). The HTS campaign identified 104 hits across 8 clusters showing blockade of hRXFP2.

The HTS output was biased for lipophilic, poorly soluble compounds. The majority of hits initially identified had a property forecast index (PFI) value greater than 6, which correlates to poor drug developability outcomes^41^. This may indicate the preference of RXFP2 to bind large, peptide-like molecules over small molecule chemicals. Removing compounds with high PFI and low efficacy (PFI > 6, < 40% inhibition of 10 nM INSL3 cAMP activity) was implemented to reduce lipophilicity. Resulting molecules were counter screened against parental HEK293 to remove compounds inhibiting cAMP with < tenfold selectivity compared to RXFP2 expressing cells. An orthogonal readout was utilized to confirm RXFP2 antagonist activity; whereby RXFP2 was transiently expressed in HEK293 stably expressing a member of the G_αq_ G protein family (murine G_α16_) to induce promiscuous G-protein coupling and subsequent calcium mobilization. This FLIPR assay detected compounds that inhibit INSL3-induced intracellular calcium (INSL3 EC_50_ = 13.44 nM).

**Figure 2:**
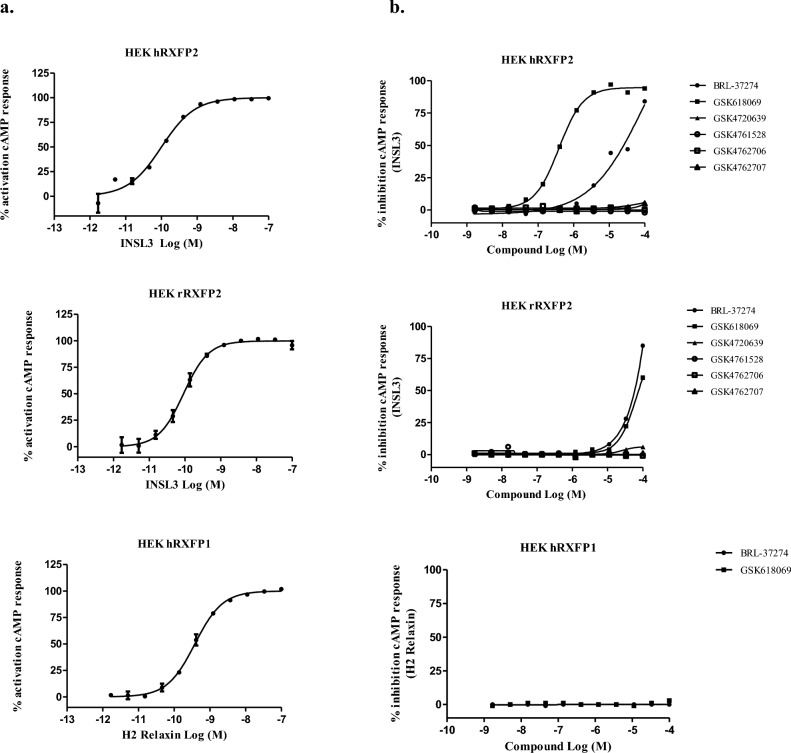
Characterization of RXFP activity assays and RXFP2 small molecule blockers in HTS and profiling assays. (**a**) RXFP2 ligands stimulate cAMP generation in cells with stable expression of a RXFP receptor. Treatment with INSL3 for 30 min dose-dependently increases cAMP in HEK293 cells with Bacmam expression of human RXFP2 (hRXFP2, n = 3) or rat RXFP2 (rRXFP2, n = 4). Treatment with relaxin (H2 Relaxin) for 30 min dose-dependently increases cAMP in HEK293 cells with Bacmam expression of human RXFP1 (n = 3). (**b**) Dose–response curves for RXFP2 antagonists in INSL3-stimulated cAMP in HEK293 cells with Bacmam expression of human RXFP2 (HEK hRXFP2) or rat RXFP2 (HEK rRXFP2). Dose–response curves for RXFP2 antagonists in relaxin-stimulated cAMP generation in HEK293 cells with Bacmam expression of human RXFP1 (HEK hRXFP1).

Active compounds (RXFP2 HTRF IC_50_ < 100 µM, RXFP2 FLIPR IC_50_ < 100 µM) were profiled to determine selectivity and rat cross-reactivity (Fig. 2). The RXFP1 assay detected compound inhibition of relaxin EC_80_-induced cAMP in HEK293 transiently expressing human RXFP1 (hRXFP1). The orthologue RXFP2 assay detected compound inhibition of INSL3 EC_80_-induced cAMP in HEK293 transiently expressing rat RXFP2 (rRXFP2; INSL3 EC_50_ = 13 nM). The compounds did not have human RXFP1 activity indicating selectivity for hRXFP2. Only 7 of 104 compounds had rRXFP2 IC_50_ < 100 µM, indicating poorly conserved receptor binding sites across species. RXFP2 blockers were organized into two potential series, based on structural similarity. A hit expansion campaign was conducted to confirm structure activity relationships, improve potency, and improve the drug-like properties (LE ~ 0.3, PFI < 6, MWt < 350, Solubility by CLND/CAD > 100 μg/mL). BRL-37274 and GSK618069 were identified as selective RXFP2 antagonists. In the HEK-hRXFP2 cAMP assay, GSK618069 demonstrated IC_50_ = 0.4 µM with 100% maximum effect; however, it had reduced potency by 200-fold against rRXFP2. BRL-37274 had IC_50_ = 15.8 µM with 91% maximum effect, and twofold less potency against rRXFP2. Ultimately, poor chemical properties and low ligand efficiency limited further compound optimization (Table 1, Supplementary Figs. S6, S7).

**Table 1:** Discovery of small molecule RXFP2 antagonists.

Compound ID	Compound structure	N hRXFP2/rRXFP2	Human RXFP2 IC_50_ (μM)	Human RXFP2% Max response	Rat RXFP2 IC_50_ (μM)	Rat RXFP2% Max Response	Human RXFP1 IC_50_ (μM)	LE	LLE (ChromLogD)	MWt	CAD SOL (μg/mL)
BRL-37274		34/36	15.8	91	31.6	87	< 4	0.3	0	366	116
GSK618069		14/10	0.4	100	79.4	56	< 4	0.3	4.6	474	> 182
GSK4761528		2/2	> 100	1.5	> 100	2	NT	NA	NA	352	> 235
GSK4720639		2/2	> 100	6	> 100	6	NT	NA	NA	399	11
GSK4762707		2/2	> 100	5	> 100	2	NT	NA	NA	522	> 209
GSK4762706		2/2	> 100	4	> 100	1	NT	NA	NA	395	> 148

Summary of RXFP2 small molecule blockers in screening and profiling assays. Table shows means of IC_50_ and max response for INSL3-stimulated cAMP activity in HEK cells with Bacmam expression of human RXFP2 (hRXFP2), rat RXFP2 (rRXFP2), or human RXFP1. N is the number of tests in the assay; LE is ligand efficiency; LLE is lipophilic ligand efficiency; MWt is molecular weight, CAD SOL is solubility; NT is not tested; NA is no result since calculation is not accurate if compound activity is > 100 uM.

### DNA-encoded library technology

To increase the diversity of RXFP2 antagonists, affinity-based DNA-Encoded Library (DEL) techniques were applied to identify compounds that bind to the entire RXFP2 ECD. The DEL campaign identified 37,563 potential binders. Prioritized compounds were resynthesized off-DNA (Supplementary Figs. S8–S11), but ECD binders were inactive in the HEK-hRXFP2 and HEK-rRXFP2 cAMP assays halting further progression (Table 1, Fig. 2). Recent models of RXFP2 predict that INSL3 binding to the ECD induces a receptor confirmation whereby the LDLa module directly interacts with extracellular regions of RXPF2 transmembrane loops to initiate receptor signaling^18,21^. The present study suggests that small molecule binding of the ECD alone is not sufficient to antagonize INSL3-induced cAMP generation.

### RXFP2 monoclonal antibody discovery

**Figure 3:**
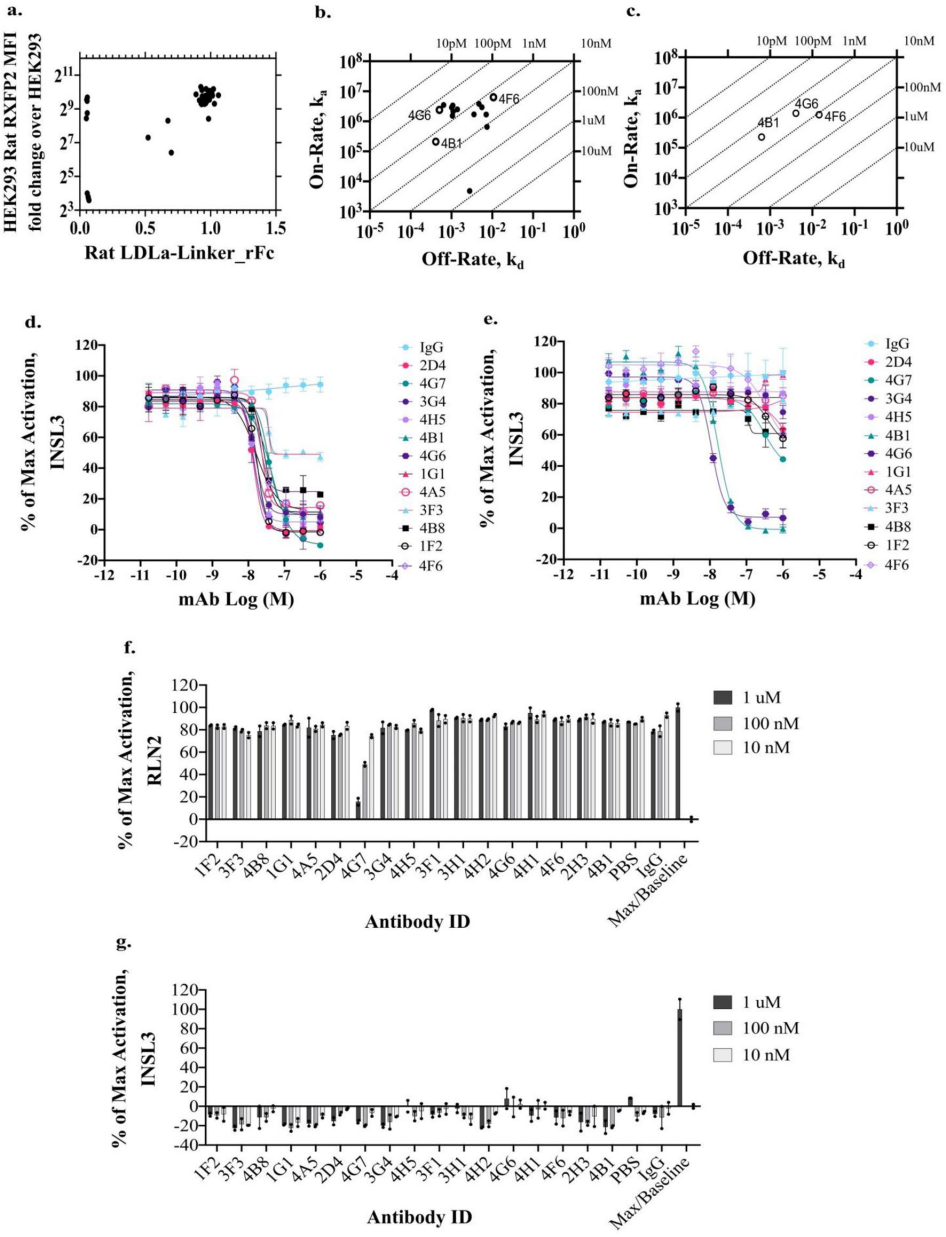
Characterization of RXFP2 mAbs in screening and profiling assays. (**a**) ELISA and cell binding data showing the binding distribution of all antibodies generated against RXFP2. The majority of antibodies generated bind to the LDLa-linker domain. (**b**) Biacore S200 ka vs kd binding data against rat LDLa-linker protein. (**c**) Biacore S200 ka vs kd binding data against human LDLa-linker protein. Three antibodies within the anti-RXFP2 panel show cross reactivity to the human LDLa-linker domain. KD values against human LDLa-linker protein: 2.75 nM (4B1), 11.7 nM (4F6), and 2.91 nM (4G6). (**d**, **e**) Representative dose–response curves of RXFP2 mAb treatment in INSL3 (EC_80_)-stimulated cAMP assay in HEK cells stably expressing rat RXFP2 (**d**, n = 2) or human RXFP2 (**e**, n = 2). The INSL3 EC_80_ of 13.96 nM and 2.92 nM were used, respectively. (**f**) Three-point RXFP2 mAb selectivity screen for activity against relaxin-stimulated cAMP activity in HEK cells stably expressing rat RXFP1 (n = 2). (**g**) Three-point RXFP2 mAb agonism screen for RXFP2 mAb activity in cAMP assay in HEK cell stably expressing rat RXFP2 (n = 2).

Monoclonal antibodies were explored as an alternate modality to neutralize RXFP2 activity. Although RXFP2 has a large ECD for antibody-based targeting, the ECD is highly conserved between mouse and rat, which might hinder a mouse immunization strategy. The sequence identity between rat and mouse for the LDLa-linker, LRR, and ECL domains are 97%, 95%, and 100%, respectively. The sequence identity between rat and human for the LDLa- linker, LRR, and ECL domains are 81%, 83%, and 87%, respectively. The Alphafold prediction models of rat LDLa-linker and LRR domains suggest that the majority of non-conserved residues are on the convex face of the LRR domain which is the non-INSL3 binding side (Supplementary Fig. S12). To account for all potential epitopes of RXFP2, a DNA immunization strategy was employed using constructs encoding the full-length rat and human RXFP2 protein in conjunction with tolerance breaking peptides^42^ and immunomodulators^43^. Using a hybridoma workflow, a panel of 47 anti-rRXFP2 antibodies was generated. Thirty-eight of the discovered antibodies bind to the LDLa-linker of the ECD (Fig. 3a). Sequencing and clonotyping revealed that the anti-RXFP2 antibodies consisted of 26 unique clonotypes. Picking one representative clone from each clonotype, 26 antibodies were recombinantly expressed in a rat IgG2b backbone to evaluate their affinities and neutralizing activities. The binding kinetics of the antibodies against rat and human LDLa-linker proteins were determined (Fig. 3b,c). The antibodies had a wide range of affinities against the rat LDLa-linker protein (Table 2). Only antibodies, 4B1, 4F6, and 4G6, had cross reactivity to the human LDLa-linker (Fig. 3c).

**Table 2:** Discovery of RXFP2 monoclonal antibodies.

mAb	Human LDLa-linker	HEK293-human RXFP2 cAMP		Rat LDLa-linker	HEK293-rat RXFP2 cAMP		H295R-rat RXFP2 CYP11B1 mRNA	H295R-rat RXFP2 CYP11B2 mRNA
ID	k_d_ (nM)	IC_50_ (nM)	% Max Response	k_d_ (nM)	IC_50_ (nM)	% Max Response	IC_50_ (nM)	IC_50_ (nM)
1F2	NB	~ 405	ND	0.69	19.1	76.1	0.32	0.10
1G1	NB	ND	ND	0.18	28	87.2	0.27	0.17
3F3	NB	ND	ND	11.4	37	78.5	3.85	0.51
4A5	NB	ND	ND	4.23	23.2	91.0	2.85	0.31
4B8	NB	~ 116	ND	0.55	21.4	86.6	0.23	0.17
2D4	NB	> 1000	ND	0.52	21.0	86.8	0.14	0.11
3F1	NB	~ 589	ND	1.24	19.9	69.7	0.27	0.23
3G4	NB	ND	ND	2.07	16.1	83.3	0.33	0.20
3H1	NB	ND	ND	1.89	15.8	71.6	0.33	0.16
4G7	NB	~ 311	ND	NB	45.5	81.9	ND	ND
4H2	NB	ND	ND	0.32	18.9	85.7	0.26	0.13
4H5	NB	ND	ND	0.35	14.2	89.2	0.21	0.15
2H3	NB	ND	ND	582	158.1	78.9	17,000	72.06
4B1	2.75	16.9	84.2	1.97	35.8	77.2	1.72	0.75
4F6	11.7	~ 354	~ 82.7	1.70	48.5	80.4	ND	ND
4G6	2.91	15.6	84.5	0.21	14.1	82.5	0.33	0.10
4H1	NB	~ 218	ND	1.01	149.7	69.7	ND	ND

Summary of RXFP2 antibody characteristics in LDL-linker binding assay, INSL3-simulated cAMP activity assays in HEK cells stably expressing either human RXFP2 or rat RXFP2, and INSL3-stimulated CYP11B1 mRNA and CYP11B2 mRNA expression in H295R stably expressing rat RXFP2. NB = No binding occurred. ND = Not determined due to insufficient data points. N = 2 per assay.

RXFP activity assays were developed in HEK293 with stable expression of RXFP2 or RXFP1. RXFP2 antagonists were identified using a primary, three-point antagonist screen at INSL3 EC_80_-induced cAMP generation in HEK293-rRXFP2 cells (INSL3 EC_50_ = 8.4 nM). Putative hits were confirmed by 11-point dose–response curve up to 1 µM mAb. Additional profiling was conducted in INSL3 EC_80_-induced cAMP generation in HEK293-hRXFP2 (EC_50_ = 2.9 nM) and relaxin EC_80_-induced cAMP generation in HEK293-rat RXPF1 (EC_50_ = 29.5 nM). See Supplementary Fig. S14for INSL3 and relaxin-induced cAMP dose responses. Seventeen antibodies were blockers of INSL3-stimulated rRXFP2 signaling and most had potencies in the double-digit nM range (Table 2, Representatives in Fig. 3d and e, Supplementary Table S2). Only two antibodies, 4G6 and 4B1, showed strong blockade of hRXFP2 (Fig. 3e). These RXFP2 mAbs were selective with no blockade of rRXFP1 except for 4G7 (Fig. 3f) and did not cause rRXFP2 agonism (Fig. 3g).

To better understand the diversity of epitopes that were covered by the RXFP2 mAbs, a bead-based high-throughput epitope binning experiment^44^ revealed 4 epitope bins across the 17 antibodies (Supplementary Fig. S13). The human/rat RXFP2 cross reactive antibodies belong to two epitope bins and were further evaluated for binding to the linker-less human LDLa peptide (UnitProt Q8WXD0 43-80) by ELISA. 4F6 and 4G6 bind to the LDLa linker-less domain while 4B1 requires the linker domain for binding (Supplementary Fig. S13). The different domain requirements for binding are consistent with results from the binning experiment showing 4F6 and 4G6 are in a separate bin from 4B1.

### INSL3 in adrenal cell assays

**Figure 4:**
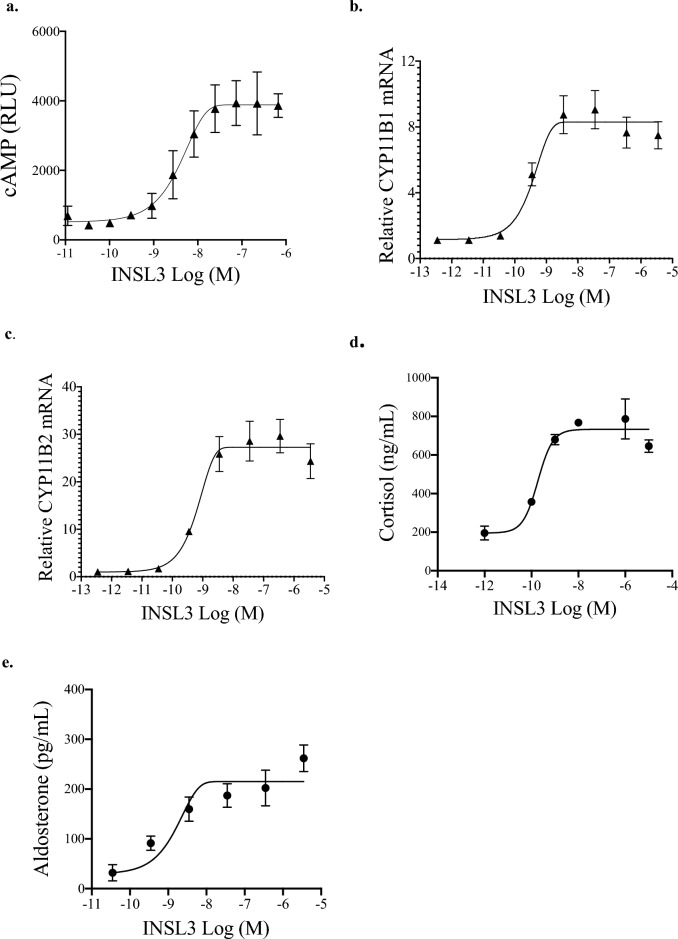
RXFP2 ligand INSL3 stimulates cAMP generation, steroidogenesis, and steroid hormone release in human adrenal cells H295R with stable expression of RXFP2. (**a**) Demonstration that treatment for 30 min with the RXFP2 ligand human INSL3 dose-dependently stimulates cAMP generation with EC_50_ = 4 nM in H295R stably expressing human RXFP2 (n = 2). (**b**, **c**) INSL3 treatment for 48 h dose-dependently increases CYP11B1 (steroid 11β-hydroxylase enzyme) and CYP11B2 (aldosterone synthase) mRNA expression levels, fold change normalized to GAPDH, in H295R stably expressing human RXFP2 (n = 3). INSL3 EC_50_ is 1.3 nM (CYP11B1) and 2.0 nM (CYP11B2). (**d**, **e**) Using ELISA detection, rat INSL3 dose-dependently increases cortisol secretion (EC_50_ = 0.16 nM) and aldosterone secretion (EC_50_ = 2.2 nM) in H295R cells stably expressing rat RXFP2 (n = 2).

Activity assays were developed using NCI-H295R, an immortalized human cell line derived from the adrenal cortex that is sensitive to cAMP stimulation and produces corticosteroids^44^. Endogenous RXFP2 mRNA determined by qPCR was too low for a robust assay; therefore, H295R with stable expression of human or rat RXFP2 were generated. The RXFP2 expression level in H295R over-expressing RXFP2 was within the range of the adrenal RXFP2 expression level in rHTN men. The potency of INSL3 on cAMP generation and the expression level of the rate-limiting enzymes for cortisol (11β-hydroxylase, CYP11B1) and aldosterone (aldosterone synthase, CYP11B2) synthesis were determined. INSL3 dose-dependently increased cAMP generation (EC_50_ = 4 nM) after 30 min of treatment. After 48 h of treatment, the EC_50_ of human INSL3 on CYP11B1 mRNA expression was 1.3 nM (H295R-hRXFP2, Fig. 4b) and 0.3 nM (H295R-rRXFP2). The EC_50_ of human INSL3 on CYP11B2 mRNA expression was 2.0 nM (H295R-hRXFP2, Fig. 4c) and 0.4 nM (H295R-rRXFP2). Cortisol and aldosterone from H295R supernatants were measured by ELISA. As shown in Fig. 4d and e, rat INSL3 EC_50_ was 0.2 nM for cortisol secretion and 2.2 nM for aldosterone secretion.

### RXFP2 antagonists in adrenal cell assays

**Figure 5:**
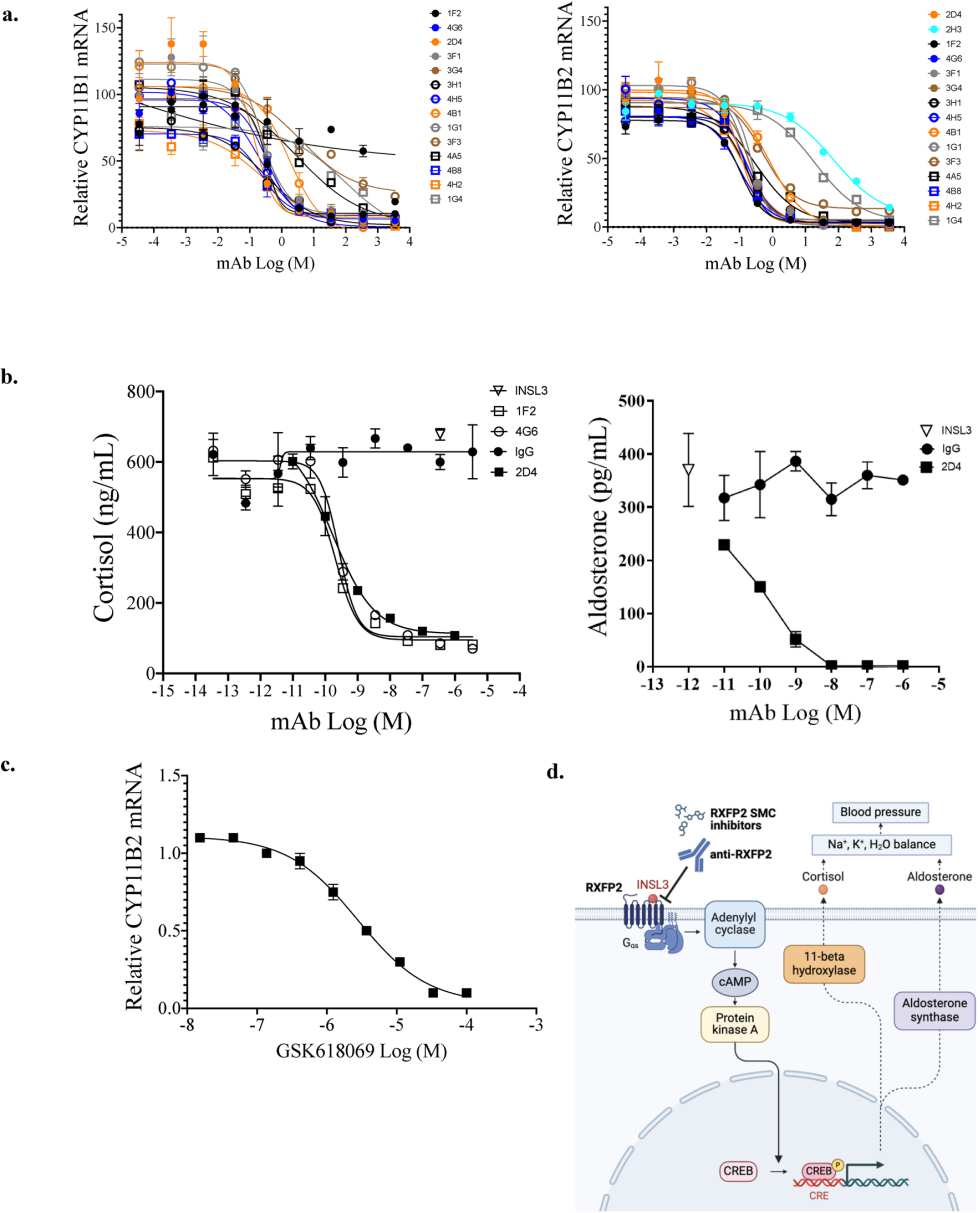
RXFP2 antagonists block INSL3-stimulated steroidogenesis and corticosteroid secretion in human adrenal cortex cells H295R stably expressing RXFP2. (**a**) Dose–response curves of RXFP2 mAb treatment in H295R cells stably expressing rat RXFP2 stimulated with 3.5 nM rat INSL3 (EC_80_) on CYP11B1 (steroid 11β-hydroxylase enzyme) and CYP11B2 (aldosterone synthase) mRNA expression levels (qPCR, relative to GAPDH control). IgG n = 6; all others n = 2. (**b**) The supernatants of cells stimulated with INSL3 and treated with the RXFP2 mAbs 1F2, 4G6, and 2D4 were assayed for cortisol (n = 2) and 2D4 was also evaluated for aldosterone (n = 2) concentration (ELISA). (**c**) Dose–response curve of RXFP2 small molecule antagonist GSK618069 in H295R cells stably expressing rat RXFP2 and stimulated with 3.5 nM rat INSL3 (EC_80_) on CYP11B2 (aldosterone synthase) mRNA expression levels (qPCR, relative to GAPDH). (**d**) Proposed hypothesis for a role of RXFP2 in causing hypertension through adrenal steroidogenesis and secretion. Graphic was created using BioRender.com. Small molecule compound (SMC).

RXFP2 antagonists were added to H295R-rRXFP2 before addition of INSL3 EC_80_ (3.5 nM) for 48 h, whereupon the cells and supernatants were collected. CYP11B1 and CYP11B2 mRNA were determined by qPCR and the RXFP2 blocker potencies were calculated (Fig. 5a). Fifteen antibodies have IC_50s_ < 50 nM, and three of the top antibodies (1F2, 4G6, and 2D4) were selected for evaluation in steroid hormone secretion assays by ELISA. 1F2, 4G6, and 2D4 dose-dependently inhibited INSL3-induced cortisol release (1F2 IC_50_ = 0.21 nM, 4G6 IC_50_ = 0.26 nM, 2D4 IC_50_ = 0.17 nM), and 2D4 inhibited INSL3-induced aldosterone release with IC_50_ = 0.05 nM (Fig. 5b) indicating functional blockade of the production of these pro-hypertensive hormones. The small molecule GSK618069 inhibited INSL3-stimulated CYP11B2 mRNA expression (IC_50_ = 2.8 µM, Fig. 5c), but it was less potent than the RXFP2 mAbs.

### RXFP2 mAb rat pharmacokinetics

**Figure 6:**
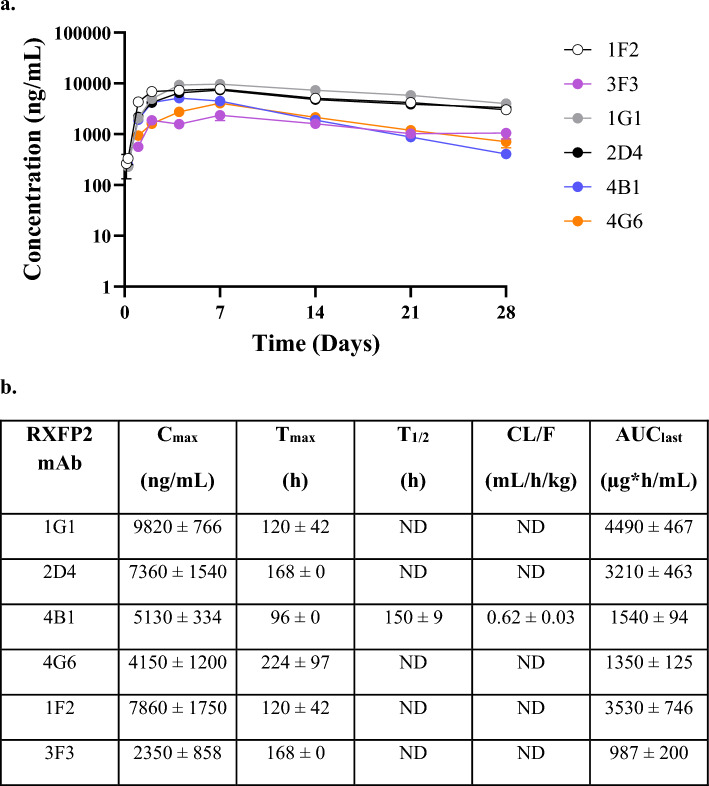
Pharmacokinetic study of RXFP2 mAbs in rat. (**a**) Six RXFP2 mAbs (1G1, 2D4, 4B1, 4G6, 1F2, 3F3) were discretely dosed to male Wistar Han rats (1 mg/kg, s.c., n = 3/mAb) once and the plasma concentration of RXFP2 mAbs was determined over 28 days. (**b**) Rat pharmacokinetic parameters calculated by non-compartmental analysis (NCA) analysis. Values are reported in mean ± standard deviation. ND: not determined due to insufficient data points or AUC extrapolated% > 20. C_max_ is the maximum concentration, T_max_ is the time to the maximum concentration, T_1/2_ is the terminal half-life, Cl/F is the apparent clearance, AUC_last_ is the area under the concentration–time profile from time zero to the time of last quantifiable concentration.

The pharmacokinetics (PK) of six RXFP2 mAbs (1G1, 2D4, 4B1, 4G6, 1F2, 3F3, 1 mg/kg, sc.) was evaluated in Wistar Han rats over 28 days. As shown in Fig. 6, the rank order of preferred PK profile was 1G1 > 2D4 > 1F2 > 4B1, 4G6, 3F3. The half-life of 4B1 was 6.25 days, and the extrapolated values for 1G1, 2D4, 4G6, 1F2, and 3F3 were ≥ 11 days. Because of their long half-lives, the clearance and volume of distribution could not be accurately determined except for 4B1, which had an apparent clearance of 0.62 ± 0.03 mL/h/kg and apparent volume of distribution of 0.133 ± 0.005 L/kg matching a typical IgG pharmacokinetic profile^46^.

## Discussion

Hypertension is a major cause of cardiovascular and renal diseases which are among the highest contributors to morbidity and mortality worldwide, and men up to 65 years old have higher prevalence of hypertension than women. Therefore, the development of new medicines for rHTN is urgently needed to reduce the health and economic burden. Complex traits such as hypertension tend to be highly polygenic and many genetic variants with small effects contribute to the phenotype. Most of the SNPs identified in GWAS for blood pressure are pleiotropic and non-coding, which makes functional mapping challenging. Thus far, approximately 30 genes with rare variants involved in blood pressure dysregulation and > 1477 common SNPs including in the RXFP2 locus have been associated with blood pressure^6,47^. In this study, we identify a novel male-specific genetic association in rHTN and provide evidence for a potential mechanism whereby the target protein, RXFP2, may regulate blood pressure. We report the first potent, selective RXFP2 antagonists for use in rodent studies.

Here, we show genetic evidence for RXFP2 as a potential mechanism for rHTN through a genome-wide association study. Sex-stratified analysis in both 23andMe and UK Biobank show the association only in men indicating that RXFP2 may play a role in the higher blood pressure in men. The shared genetic signal between the rHTN GWAS and the RXFP2 eQTL in the adrenal gland suggest a RXFP2 antagonist would be beneficial for the treatment of rHTN. Experimentally, we validated the variant to gene analysis and show that RXFP2 gene expression is increased in adrenal gland of men with hypertension. Phenome-wide significant associations of the GWAS lead SNP with hyperaldosteronism and other hypertension-related diseases further strengthen the hypothesis for a role of RXFP2 in rHTN.

Human adrenal cell assays were established to assess the functional consequences of RXFP2 agonism and antagonism. Because endogenous RXFP2 gene expression is low in H295R, they were engineered to stably express RXFP2 to increase the signal window across assays. The results show that INSL3, the natural ligand of RXFP2, increases cAMP generation in human adrenal cells, which has been previously associated with increased steroid secretion^48^. Because INSL3 also increases CRE-dependent gene transcription ^23^, steroidogenesis was evaluated in H295R stably expressing RXFP2. INSL3 dose-dependently increased 11β-hydroxylase and aldosterone synthase transcripts which are the most proximal, rate-limiting enzymes in the synthesis of cortisol and aldosterone, respectively. The impact on steroidogenesis is consistent with previously demonstrated INSL3-stimulated CYP17A1 gene expression in theca cells, which was blocked by siRNA targeted at RXFP2^15^. Further functional evaluation of RXFP2 agonism was necessary to translate the increase in gene transcription to a clinically meaningful effect. Indeed, elevations in circulating concentrations of aldosterone and cortisol are required for the hormones to achieve their pro-hypertensive effects via activation of the mineralocorticoid receptor in target tissues like kidney and brain. Thus, ELISAs were established to measure aldosterone and cortisol in supernatants from H295R stably expressing RXFP2, and INSL3 dose-dependently increased aldosterone and cortisol release. The increase in steroid hormone secretion is consistent with dibutyryl-cAMP-stimulated aldosterone and cortisol secretion in H295R^48^ and INSL3-stimulated androgen secretion in Leydig^14^ and theca cells^15^.

Since RXFP2 increased adrenal corticosteroids, it may not be surprising to find phenome-wide significant associations of rs2146377 in hyperaldosteronism and rs1535532 in primary aldosteronism^17^, which are associated with hypernatremia, hypokalemia, and hypertension. rs1535532 was one of the top eQTLs for RXFP2 in the adrenal gland and the PheWAS with primary aldosteronism was specific to men^17^. Whether adrenal RXFP2 also causes hypertension through PLC/PKC-medicated increases in aldosterone^49,50^ or through catecholamine production remains to be elucidated.

A limitation of GWAS is the possibility that associations are false-positives due to population stratification; however, the risk was mitigated by correction for primary axes of genetic variation and replication from an independent cohort. Observational studies in biobanks have the potential for healthy volunteer bias and rHTN may reflect a lack of compliance to medications, leading to possible attenuation in effect size. These limitations underscore the importance of validation and follow-up with functional assays, as was done in this study.

Discovering small molecule antagonists of class A GPCRs such as RXFP2 can be challenging, although there is an example of a RXFP2 agonist^28^. Therefore, we conducted a HTS and a monoclonal antibody campaign to discover potentially both short-acting and long-acting RXFP2 antagonists. The primary assay utilized cAMP generation as a measure of receptor activity in HEK293 cells transiently or stably expressing hRXFP2, an approach similar to what was used in the discovery campaign for RXFP2 agonists^28^. We further enhanced the ability to identify small molecule blockers by application of a DNA encoded library technology. Despite identifying modestly potent and selective RXFP2 small molecule blockers that reduce aldosterone synthase transcript in human adrenal cells, physiochemical properties including ligand efficiency and high lipophilicity limited their drug development opportunity.

The antibody discovery campaign discovered more potent and selective antagonists of both rat and human RXFP2. Some of the RXFP2 mAbs have potencies in the double digit nanomolar range that provide ≥ 85% inhibition of the INSL3-induced cAMP generation in HEK-hRXFP2 and HEK-rRXFP2. Most of them also completely inhibit INSL3-induced gene expression of 11β hydroxylase and aldosterone synthase and representative mAbs also reduce cortisol and aldosterone secretion with similar potency and efficacy as their steroidogenesis effects. The potencies of the RXFP2 mAbs in the adrenal cell assays are greater than in the HEK cAMP assay which may be related to the efficiency of the adrenal cell response, which is supported by the higher INSL3 potency in adrenal cells than in HEK. Across all assays, the RXFP2 mAbs are more potent and efficacious than the small molecule RXFP2 antagonists, which may reflect differences in binding domains.

The RXFP2 blocking antibodies bind to the LDLa-linker domain of RXFP2. Previous studies have highlighted the importance of both the linker and LDLa domains in RXFP2 receptor activation and signaling^9,18,20–23^. Removal of the LDLa results in abolished signaling^9,18,23^. Similarly, residues within the linker, GDxxGWxxxF (G46-F55), are required for receptor activation^20,21^. The current data further reinforces the critical role the LDLa-linker domain plays in receptor activation and provides insight into the possible mechanism of action of these novel RXFP2 mAbs.

We propose the RXFP2 mAbs function by interfering with regions critical for receptor activation; however, they use different mechanisms to achieve their antagonistic activity. The human LDLa peptide ELISA and epitope binning facilitated stratification of the 3 cross-reactive human/rat RXFP2 blocking antibodies into two distinct epitope bins (Supplementary Fig. S13). Antibody 4B1 requires the linker for binding; whereas, 4F6 and 4G6 require the LDLa domain for binding. To interrogate the epitopes, AlphaFold, which can accurately predict some antibody:antigen complexes, was employed^51^. High confidence predictions for 4B1 and 4G6 in complex with the rat LDLa-linker domain were obtained, but not for 4F6 (see "Methods" section for details). The structural model of the 4B1 rat LDLa-linker domain complex agrees with the experimental ELISA data, as it shows 4B1 binding to linker at the conserved GDxxGWxxxF motif (Supplementary Fig. S15). This motif has been shown to be crucial for receptor activation, likely by allowing rearrangement of the linker to allow the LDLa domain to interact with the extracellular loops of the transmembrane domain^20,21^. 4B1 likely blocks receptor activation indirectly by preventing linker rearrangement. The AlphaFold model for the 4G6 rat LDLa-linker complex is also in agreement with the experimental data and predicts that the 4G6 epitope is mostly located on the LDLa domain (Supplementary Fig. S15). The predicted epitope covers the N-terminus of the first beta-sheet (K8-F11) which has been shown to be crucial for receptor activity^23^. The structural model suggests that 4G6, unlike 4B1, may directly block the interaction between the LDLa domain and the transmembrane domain, which is required for receptor signaling. LDLa domains of RXFP1 and RXFP2 modulate receptor activation via similar mechanisms^52^. However, 4G6 did not block relaxin-induced RXFP1-mediated cAMP generation and chimeric RXFP1/2-mediated activity was not evaluated.

The levels of RXFP2 mAbs that are required for blocking adrenal hormone release are likely achievable in rodents based on pharmacokinetic (PK) studies in rat. Using the mAb concentration at trough, we assumed dose-linearity to estimate the dose and length of time to achieve tenfold higher IC_50_ HEK-rRXFP2 cAMP activity, which is higher than what would be required to achieve the IC_50_ in the H295R-rRXFP2 steroid hormone assays. 2D4 at approximately 9 mg/kg, 1G1 or 1F2 at approximately 10 mg/kg, 4B1 at approximately 1324 mg/kg, and 4G6 at approximately 30 mg/kg could achieve that inhibition for approximately 28 days in rat.

Given the expression of RXFP2 in organs that are important in the regulation of blood pressure, and the previously described functions of INSL3/RXFP2 in the brain^16^, renal glomerular cells^11^, and androgen secretion^14^, we cannot rule out non-adrenal mechanisms for RXFP2 in the regulation of blood pressure. During development, androgens increase RXFP2 expression in gubernaculum which mediates testis descent^53^. Whether androgens increase RXPF2 in adult tissues and is a mechanism for the sex-specific difference in hypertension remains unknown. Additionally, RXFP2 signaling does not initiate β-arrestin-mediated internalization^54^ so the lack of desensitization may contribute to rHTN. With the discovery of potent, selective RXFP2 antagonists with good PK, the broader contributions of RXFP2 could be further explored.

Our goal for conducting GWAS in resistant hypertensive individuals was to identify new drug targets and ultimately new medicines for the treatment of rHTN because of its critical importance in the development and progression of CVD and CKD. Direct inhibition of aldosterone synthase lowers blood pressure in rHTN^55^. Whether greater blood pressure reduction could be achieved by blocking RXFP2 which targets multiple mechanisms remains to be tested. Based on historical data and statistical modeling, drug targets with human genetic evidence are 2.6 times more likely to be successful during clinical trials^56^. The current studies provide new genetic evidence in rHTN in men and a role for adrenal RXFP2 activity in rHTN. Whether RXFP2 activity is causal or secondary to rHTN remains to be determined. With the discovery of these RXFP2 antagonists, we have tools to further interrogate the role of RXFP2 in health and disease and to provide promise for developing genetically informed drug treatments for precision medicine in rHTN.

## Methods

### GWAS and PheWAS

#### 23andMe

GWAS and PheWAS were conducted on data from consented European 23andMe research participants. Participants provided informed consent and volunteered to participate in the research online, under a protocol approved by the external AAHRPP-accredited IRB, Ethical & Independent (E&I) Review Services. As of 2022, E&I Review Services is part of Salus IRB (www.versiticlinicaltrials.org/salusirb). A maximum set of unrelated individuals (IBD sharing < 700 cM) were used in association analyses. Variants were imputed with Minimac3, using 1000 genomes Phase 3, UK10K, and the HRC as reference panels. The association was conducted by fitting a logistic regression model with age, sex, five genetic PCs, and genotyping platforms as covariates. *p* values are those from a likelihood ratio test. Sex-stratified analyses adjusted for age, genetic PCs and genotyping platforms in the model. LD-score regression intercept was used to correct for inflation before reporting of GWAS results. PheWAS was conducted over a total of 1122 phenotypes, a Bonferroni significance threshold of 0.05/1122 was used.

#### UK biobank

*Phenotyping.* Cases of treatment-rHTN were defined as participants that meet all of the following: (1) self-reported ≥ 4 hypertension medications at baseline assessment and (2) self-reported hypertension, systolic blood pressure ≥ 140 mmHg, or diastolic BP ≥ 90 mmHg at baseline assessment. Controls for non-treatment-rHTN were defined as participants that met any of the following: (1) self-reported hypertension and ≥ 1 and ≤ 3 hypertension medications; (2) systolic blood pressure ≥ 140 mmHg; or, (3) diastolic BP ≥ 90 mmHg at baseline. Controls with no hypertension were defined as all UK Biobank participants that did not have any of the following: (1) self-reported hypertension and 1 + hypertension medications; (2) systolic blood pressure ≥ 140 mmHg; or, (3) diastolic BP ≥ 90 mm at baseline. Sex was defined according to UK Biobank Field 22001 (biobank.ndph.ox.ac.uk/showcase/field.cgi?id=22001). European ancestry was defined according to UK Biobank Field #22006 (biobank.ctsu.ox.ac.uk/crystal/field.cgi?id=22006). We excluded all third degree relatives (KING > 0.8). UK Biobank data was accessed using application 20,361 and all of the participants provided informed consent.

*Genetic analysis.* Genotyping, quality control, and imputation were performed centrally by UK Biobank. Among the UK Biobank study population, we filtered the genotype data to only include variants with MAF > 1%, INFO score > 0.8, and Hardy–Weinberg equilibrium exact test *p* value greater than 1e-6. We required a maximum per-variant and per-sample missing call rate < 0.1. The final target dataset included 9.4 million SNPs after excluding all duplicate SNPs. Primary analyses were run using logistic regression in the hail software package comparing cases of treatment-rHTN to controls with non-treatment-rHTN. Secondary analyses were run, also using logistic regression in the hail software package, comparing cases of treatment-rHTN to controls with no hypertension. Both primary and secondary analyses were stratified by sex as defined by UK Biobank.

*PheWAS using publicly available data.* Phenotyping and genetic analysis methods for the publicly available UK Biobank PheWAS are available at www.nealelab.is/blog/2017/9/11/details-and-considerations-of-the-uk-biobank-gwas. All PheWAS results for rs2146377 are available at https//pheweb.org/UKB-Neale/variant/13:32179063-G-A.

#### FinnGen

Phenotyping and genetic analysis methods for the FinnGen cohort have been previously published and all data used in this manuscript are publicly available at https://r9.finngen.fi. All PheWAS results for rs2146377 are available at https://r9.finngen.fi/variant/13:31631505-A-G. We want to acknowledge the participants and the investigators of the FinnGen study.

#### GTEx eQTL data

The data used for the eQTL analyses described in this manuscript were obtained from the GTEx Portal on October 21, 2023 and/or dbGaP accession number phs000424.v8.p2.

### Gene expression analysis in adrenal gland

Frozen adrenal gland tissues from six hypertensive and six normotensive men were acquired under the relevant guidelines and regulations from BioIVT and used in accordance with the corresponding institutional review board approved informed consent form. Tissues were dissociated using GentleMACS M Tubes, followed by total RNA isolation (Qiagen), and cDNA synthesis (Applied Biosystems). qPCR was completed using TaqMan probes for RXFP2 (Hs00373128_m1), CHGB (Hs01084631_m1), NOV (Hs00159631_m1), and ACTB (Hs99999903_m1). Taqman probes for the adrenal medulla (CHGB) and cortex (NOV) were used to rule out regional bias of samples. For the human adrenal tissue RXFP2 transcript analysis, power analysis was determined from a pilot study for sample size determination. To minimize potential sources of bias, samples for qPCR assay were randomized and blinded and results were tested for normality (GraphPad Prism). Based on the distribution of results, the Mann–Whitney U test was applied and *p* < 0.05 indicated statistical significance.

### RXFP Bacmam expression in HEK293

The nucleotide sequences used in Bacmam transfections encoded RXFP protein as described in UniProt (Q9HBX9. RXFP1_human; Q8WXD0. RXFP2_human; Q5ECL0. RXFP2_rat). HEK293- MSRII cells were maintained in DMEM‐F12, supplemented with 10% fetal bovine serum (FBS) and L-glutamine at 37 °C/5% CO_2_. HEK293 stably expressing G_α16_ were maintained in DMEM‐F12, supplemented with 10% FBS, L-glutamine and selection antibiotics at 37 °C/5% CO_2_. Transient receptor expression was achieved using modified baculoviruses (BacMam), generated according to manufacturer’s instructions using the Bac-to-Bac system (Invitrogen, UK) encoding hRXFP1, hRXFP2 or rRXFP2. Cells were transduced with RXFP-BacMam at an optimized multiplicity of infection (MOI) of 5.

### HEK-RXFP screening and profiling assays

cAMP generation was measured using the Lance Ultra cAMP kit as per manufacturer’s instructions (Perkin Elmer) in HEK293 cells transduced in tissue culture flasks with RXFP BacMam, incubated at 37 °C/5% CO_2_ for 18 h before assay. Primary single shot HTS and specificity assays were performed in 1536-well format. HEK293 wild type (wt) or RXFP2-transduced HEK293 cells were detached, washed in PBS and resuspended in assay buffer (HBSS, 5 mM Hepes, 0.1% BSA, 0.5 mM IBMX, pH 7.4). The cell solution was plated at a density of 500 cells/well in 3 µL into white, 1536-well plates pre-stamped with 50 nL of test compound and incubated for 30 min. Agonist stimulation was induced with the addition of a further 2 µL of assay buffer supplemented with a final EC_80_ concentration of ligand (20 nM Isoprenaline- no RXFP receptor or 4.5 pM INSL3- RXFP receptor). After a 30 min incubation, 4 µL of Lance detection reagent was added and the FRET signal read 1 h later using a TR-FRET-capable plate reader, with excitation at 340 nm and emission at 615 nm and 665 nm. Results were expressed as acceptor fluorescence (signal at 665 nm/signal at 615 nm) normalized to 100% inhibition and 0% inhibition controls (+/− agonist stimulation respectively). Further confirmation, selectivity and ortholog cAMP assays were performed post-HTS in 384-well format using a modified protocol. Here, 10,000 cells/well were plated in 5 µL into white, 384-well plates pre-stamped with 100 nL of test compound and incubated for 30 min. Agonist stimulation was induced with the addition of a further 5 µL of assay buffer supplemented with a final EC_80_ concentration of ligand (120 nM INSL3- RXFP2, 0.89 nM INSL3- rRXFP2, 1.6 nM H2 Relaxin- RXFP1). After a 30 min incubation, 10 µL of Lance detection reagent was added and results collected as above.

For the RXFP2 orthologue assay, calcium detection was measured using FLIPR assay technology in HEK293‐ MSRII G_α16_ cells. Cells were resuspended in fresh cell culture media in the presence of RXFP2 BacMam and seeded at a density of 15,000 cells/well in 50 µL into black clear‐bottomed 384‐well plates and incubated at 37 °C/5% CO_2_ for 24 h prior to assay. Media from wells was aspirated and replaced with 30 µL/well of assay buffer (HBSS, 25 mM HEPES, pH 7.4) supplemented with dye-loading reagents (30 µM probenecid, 3 µM Fluo‐4 and 750 µM Brilliant Black). Plates were incubated for 1 h at 37 °C/5% CO_2_. Test compounds were added to dye-loaded cells at 10 µL/well and incubated for 10 min. Agonist stimulation was induced with the addition of 10 µL/well assay buffer supplemented with a final EC_80_ concentration of ligand (2.4 nM INSL3). Fluorescence was detected immediately using a FLIPR TETRA plate reader, with excitation at 488 nm and emission at 530 nm. Results were expressed as fluorescence (signal at 530 nm-background read) normalized to 100% inhibition and 0% inhibition controls (−/+ agonist stimulation, respectively). Human INSL3 (Phoenix Pharmaceuticals Inc, 035-27) was used in screening and profiling assays. Dose curve fitting and IC_50_ determinations were performed in Activitybase XE (version 9.2, IDBS) using 4-parameter logistic curve (Model 203).

### DNA encoded library selection

DEL pools containing 93 libraries were used to screen against the following RXFP2 constructs: human LDLa linker [huRXFP2-LDLa-linker(38-105)-ratFc-8XHIS] and rat RFXP2 LDLa linker [ratRXFP2-LDLa-linker(20-88)-ratFc-8XHIS]. Selections were performed in triplicate. In the first rounds of selection, DEL molecules were enriched against their respective target. In the final round of selection, the enriched DEL molecules were split equally across 3 selection conditions: (1) human LDLa-linker_rat Fc or rat LDL-linker_rat Fc, (2) rat Fc (control for LDLa_linker), and (3) buffer only. In a round of selection, the DEL pool was incubated with 1 mM of protein at 25 °C for 45 min in 100 µL of selection buffer (25 mM Tris, pH 7.4, 150 mM NaCl, 2.5 mM CaCl_2_, 0.10% Triton X-100, 10 mM Imidazole, 0.1 mg/mL ssDNA). Following incubation, the protein was captured on 50 µL slurry of IMAC magnetic beads (equal volumes of Invitrogen Dynabeads His Tag slurry and Pierce Ni-NTA magnetic bead slurry) pre-washed with selection buffer. While bound to the magnetic beads, the samples were washed twice with selection buffer. Following the washes, beads were re-suspended in 60 µL of elution buffer (25 mM Tris, pH 7.4, 150 mM NaCl, 2.5 mM CaCl_2_, 0.10% Triton X-100, 10 mM Imidazole). In all but the last round, a heat elution step (95 °C for 10 min) was performed to recover the target bound molecules before starting the next selection round with fresh protein. Recovered DEL samples were analyzed using SYBR Green qPCR on a Roche LightCycler in order to determine how many PCR amplification cycles were required for DNA sequencing. Following the last round of selection, heat elution was not performed. Samples on IMAC resin were resuspended in molecular biology grade water and amplified by PCR. PCR reactions were cleaned up with AMPure resin using 1.8× equivalents of resin to PCR product. Sequencing was performed on an Illumina Novaseq.

Selections with the extracellular domain construct (TEV-Flag-6XHis-HiBit-homo sapiens RXFP2) were performed with DEL pools containing 98 libraries pooled with equal copies of every small molecule. Triplicate selections were performed as described above in selection buffer containing 50 mM potassium phosphate pH 7.4, 300 mM NaCl, 1 mM CHAPS, 10 mM imidazole, 0.1 mg/mL ssDNA. Selections were repeated in the same buffer with the addition of 5% inositol. Elution was performed in buffer containing 50 mM potassium phosphate, pH 7.4, 300 mM NaCl, 1 mM CHAPS, and 10 mM imidazole. After selection, enriched DEL molecules were PCR amplified, AMPure treated, quantified, and sequenced as described above.

### Hybridoma development

Spleens and lymph nodes were harvested from DNA immunized mice three days following the final boost. Final boosts for mice was either 25 µg each hu and rat LDLa-linker-rFc or irradiated rRXFP2 HEK293 stable cells (Rad Source RS-2000). Splenocytes and lymphocytes from immunized mice were fused with SP2/mIL-6 mouse myeloma cells (American Type Culture Collection CRL-2016) by electrofusion (Nepagene ECFG21) and incubated at 37°C in the presence of 5% CO_2_ overnight in ClonaCell-HY Medium C (Stemcell 03803). The following day the cells were harvested and prepared for plating into ClonaCell-HY Medium D (Stemcell 03804). Each 45 million fused lymphocytes was incubated with 1.0 mg of 0.2 µm syringe filtered sodium azide free anti-mouse IgG Fc fragment specific secondary-FITC reagent (Jackson Immunoresearch Cat#115-096-071), 10 mL total volume. The mixture was incubated for 5 min in the dark at room temperature. The 10 mL volume was then combined with 90 mL of ClonaCell-HY Medium D. The mixture was then shaken well and incubated for 5 min incubation, in the dark, at 37 °C. Finally, 100 mL of the mixture was plated into 6 omnitray plates (Nunc 140156) and hybridoma colonies were allowed to grow for ~ 7 days post fusion after which the omnitray plates were imaged on the Clonepix 2 colony picker (Molecular Devices) and the IgG + hybridomas were picked into 96-well plates containing 200 µL/well of ClonaCell-HY Medium E (Stemcell 03805). The IgG + hybridomas were allowed to grow in 96-well plates for ~ 7 days, upon which the supernatants were screened for RXFP2 specificity on a flow cytometer (CytoFlex LX).

### Flow cytometry analysis of hybridoma clones

Sera from Balb/c and Swiss-Webster mice was taken four days following the fourth, sixth, eighth, eleventh, and thirteenth injection of RXFP2 DNA and screened on HEK293 parental, HEK293-rRXFP2, HEK293-hRXFP2. Hybridoma supernatants and purified antibodies were screened on HEK293 parental, HEK293-rRXFP2, and HEK293- hRXFP2. Cells were dissociated from flasks using non-enzymatic Corning CellStripper (25-056-C), resuspended in media, counted, centrifuged at 1200 rpm for 5 min, washed with 1× PBS, and added to 96-well U-bottom plates (Corning 3365) at 500,000–1,000,000 cells per well. Sera were diluted 1:100 and then serially diluted 1:2 in PBS containing 5% FBS (R&D Systems S12450H); hybridoma supernatants were screened neat. Samples were added to cells (50 µL/well) and incubated on ice for 60 min. Plates were centrifuged (1200 rpm, 5 min, 4 °C) and washed three times with PBS/5% FBS (200 µL/well). APC-Goat mouse IgG Fc fragment specific (Jackson Immunoresearch 115-136-071; 1:100 diluted in PBS) was then added and the plates incubated on ice in the dark for 60 min. Plates were again centrifuged and washed three times with PBS/5% FBS (200 µL/well), and a final resuspension of 150 µL/well of PBS was performed. The plates were then read using a Cytoflex LX flow cytometry (Beckman Coulter). Median fluorescence intensity (MFI) of each sample was then measured using the FlowJo software (Treestar, Inc.).

### ELISA for antibody screening

Rat LDLa-linker_ratFc, human LDLa-linker_ratFc, or ratFc, or human LDLa peptide (Q8WXD0 43-80) was diluted to 1 µg/mL in 1X PBS and immobilized in separate 96-well ELISA plates (Nunc Maxisorp), 50 µL/well. The plates were then incubated overnight at 4 °C. The following day the plates were washed three times with ELISA wash buffer (PBS + 0.05% Tween-20) and then blocked with ELISA blocking buffer (PBS + 0.05% Tween-20 + 1% BSA), 200 µL/well, and incubated 1 h at room temperature on a shaker. Plates were again washed three times followed by the addition of the primary antibodies. Hybridoma supernatant was added neat, 50 µL/well; mouse serum was added 1:100 and then serially diluted 1:2 for 8 points, 50 µL/well. Purified antibodies were added 5 µg/mL in the first well and serially diluted down the plate 1:2 for 8 points, 50 µL/well. Primary antibody was incubated for 1 h at room temperature on a shaker. Plates were again washed three times followed by the addition of the secondary antibody; HRP-Goat anti mouse Fab2 fragment specific antibody (Jackson Immunoresearch, 115-036-006) or HRP-Goat anti rat Fab2 fragment specific antibody (Jackson Immunoresearch 112-036-072) was added to the plates 1:5000, 50 µL/well, incubated 1 h at room temperature on a shaker. Plates were washed a final three times and Turbo-TMB ELISA (Thermo 34022) reagent was added, 50 µL/well, incubated 10 min at room temperature on a shaker. Stopping solution (2N Sulfuric acid) was added to the plates 50 µL/well, and then read on a plate reader (SpectraMax Molecular Devices) at 450 nm.

### In vitro antibody epitope binning

Biotinylated Rat LDLa-linker_ratFc protein was captured at a concentration of 2 µg/mL to lumavidin beads (Luminex). Capture was performed for 1 h at room temperature in the dark. Beads were then washed 2× with flow buffer (1× PBS + 2% FBS) by centrifugation 3 min at 3500 rpm; wash buffer was aspirated and beads were diluted to a concentration of 2.5 × 10^6^ beads/mL. Protein coated beads were then incubated with each antibody in the panel (herein referred to as benchmark) at 5 µg/mL of antibody with 1.25 × 10^6^ beads/mL for 45 min at room temperature in the dark (one benchmark mAb per lumavidin bead type). Beads were again washed 2 × with flow buffer by centrifugation. After wash all bead types were pooled and made to 1.25 × 10^6^ beads/mL. All test panel antibodies were then prepared in a 96-well v-bottom plate, 20 µL/well of antibody at 3.3 µg/mL concentration. The pooled beads were then added to the v-bottom plate, 40 µL of pooled beads per well and then incubated with the test panel antibodies for 45 min at room temperature in the dark. Beads were again washed 2 × with flow buffer by centrifugation. Detection antibody (Jackson Immunoresearch, G anti Ms-FITC) was then added at 5 µg/mL, 50 µL/well for 15 min at room temperature in the dark. Beads were again washed 2× with flow buffer by centrifugation. Final resuspension of beads was performed with 50 µL of flow buffer and then plates were read.

### AlphaFold multimer prediction

AlphaFold multimer v2.2.4 (doi.org/10.1101/2021.10.04.463034) was used to generate a prediction of antibody:rat LDLa-linker (Uniprot entry: Q5ECL0 residues 19-88) complex for 4B1, 4F6, and 4G6. Twenty-five predictions were generated for each antibody:antigen complex, and model quality was evaluated based on their respective iPTM scores. Previous work ^57^ has shown accurate antibody:antigen complex predictions are possible and an interface predicted modeling score (iPTM) score threshold of 0.75 corresponds to a possible high confidence model cutoff. While we obtained an iPTM score of 0.76 and 0.82 for the 4B1:antigen and 4G6:antigen complex, respectively, no prediction of the 4F6:antigen reached the iPTM threshold. The top iPTM scoring models for 4B1 and 4G6 were further evaluated and visualized using PyMOL 2.5.5 (Schrodinger, Inc.).

### Generation of recombinant proteins and IgG purification

Heavy and light chain variable domains for the antibodies of interest were cloned into in-house constructs and prepared as an endo-free maxi-scale DNA prep (Genewiz). Heavy and light chain cDNA was mixed in a 1:2 ratio and utilized to transfect Expi293 cells according to the manufacturer’s guidelines (ThermoFisher). Enhancers were added to the culture 16–18 h post transfection (Gibco), and the culture was allowed to grow for 6 days. After 6 days the cultures were harvested, spun down at 300 g for 10 min, transferred to clean tubes, and spun down again at 10,000 g for 10 min. Transfection supernatant was collected in clean 50 mL falcon tubes. Supernatant from each transfection was incubated overnight at 4 °C with 1× PBS washed Protein G (Pierce). The following day the supernatant + protein G mixtures were added to 20 mL econo-pac gravity flow columns (BioRad). Flow through was collected in 50 mL falcon tubes and saved at 4 °C. The columns were washed with 5 column volumes of 1X PBS. Finally, the columns were eluted with 4 column volumes of elution buffer (0.1 M Acetic Acid/0.15 M NaCl). The eluate was collected in 15 mL falcon tubes and neutralized with 1 column volume of 1 M MOPS pH = 7. Buffer exchange to 1X PBS was performed in 50,000 MWCO Amicon Ultra-15 centrifugal filters (Millipore). The purity of the purified antibodies was evaluated using HPLC (mAb purities > 95%) and the sequences are shown in Supplementary Table S2.

Additional proteins were generated for use in the RXFP2 antibody discovery campaign. Plasmids for human or rat LDLa-linker were constructed to express the LDLa-linker domain followed by an enterokinase cleave site, a rat Fc domain, and an 8xHis purification tag. Briefly, Expi293 cells were transfected with each expression plasmid separately and harvested after 4 days of culture. Histrap excel was used to purify each protein followed by SEC polish and QC (SDS-PAGE, endotoxin < 0.4 EU/mL). Enterokinase digestion was used to cleave the LDLa-linker from the Fc tag and separation was carried out using Hispur Ni-TA resin batching binding. This was followed by Superdex 200 cleanup. Additionally, an expression plasmid was generated to encode the extracellular domain of hRXFP2 (a.a. 238-360) followed by the hagfish variable lymphocyte receptor B (VLRB) domain (a.a. 133-200), TEV, and the purification tags Flag, Hisx6, and HiBit. The VLRB was used to cap the C-terminal end of the native RXFP2 LRR domain which resulted in aggregation during purification. This hybrid strategy replaced the hydrophobic LxxLxLxxN region with LxxLxLASN. The purity of the recombinant proteins was evaluated using HPLC (human LDLa-linker-Fc and rat LDLa-linker-F purities are 100%) and the sequences are shown in Supplementary Fig. S16.

### Biacore for mAb affinity determination

Goat anti Rat Fc gamma fragment specific (Jackson Immunoresearch, 112-005-071) was immobilized at 30 µg/mL to a Series S CM5 chip (Cytiva Life Sciences). In three separate runs, twenty different purified recombinant rat anti RXFP2 antibodies were then captured to the CM5 chip at a concentration of 5 µg/mL. The antibody panel was captured; rat LDLa-linker or human LDLa-linker protein was flowed as analyte at the following concentrations: 20 nM, 10 nM, 5 nM, 2.5 nM, 1.75 nM, and 0 nM. Antibody kinetics were evaluated using a 1:1 fit model (Biacore S200).

### Hybridoma sequencing

Hybridoma cells cultured to confluency in 96 wells (about 5 × 10^4^ cells/well) were lysed using lysis/binding buffer (Ambicon, 61012). RNA reverse transcription and cDNA synthesis were carried out using Dynabeads mRNA Direct Micro kit (Ambicon, 61012).

Variable light (VL) and variable heavy (VH) domains were amplified separately from the cDNA using touchdown PCR, KOD Hot Start DNA polymerase (Novagen, 71842) and previously described (Ref PMID 29357282) pooled heavy and light chain primers annealing to leader sequence of the variable domains as well as the CH1 region of the IgG domain and the CL region of kappa light chains, respectively. The PCR product was cleaned up in individual wells using SPRI (AmPure XP) beads (Beckman Coulter, A63881). The purified DNA was quantified using Qubit 4 Fluorometer (Thermo scientific). Illumina sequencing barcodes and adaptors were added to the amplicons by a second PCR using the Nextera XT Index Kit v2 (Illumina, FC-131-2001) and the KOD Hot Start DNA polymerase (Novagen, 71842). The PCR product was cleaned up in individual well using SPRI (AmPure XP) beads (Beckman Coulter, A63881) and quantified using PicoGreen (Thermo Fisher) + TECAN spark. The final sequencing library was generated by combining 5 ng per well of PCR reactions into one single tube. The DNA was quantified and normalized to 1 nM. The library was sequenced on Illumina Miseq sequencer using the 2 × 300 bp amplicon protocol.

After sequencing, individual samples were de-multiplexed using the sample barcodes and paired sequence reads were aligned to generate a contig per sequence read pair. Heavy and light chain variable domains were assigned to the contig sequences. Next, one count table containing the most frequent variable regions for each sample was generated, where the top ranked sequence of each sample was assumed to be the VH or VL region of the hybridoma clone from which the individual sample originated. Finally, the hybridoma clones were grouped into “clonotypes” based on the assigned VH germline sequence, the length of the CDR-H3, and CDR-H3 sequence similarity.

### Generation of RXFP stable expression in H295R and HEK293

Stable NCI-H295R adrenal cell clones expressing hRXFP2, rRXFP2, or rat RXFP1 were generated and cultured in DMEM:F12 (Gibco) supplemented with 2.5% NuSerum, 1× Pen-Strep, and 1× insulin-transferrin-selenium-ethanolamine (ITS-X). Briefly, Lenti-X HEK293T cells were maintained in antibiotic free media and transfected (Lipofectamine LTX) with plasmids encoding rat and human RXFP1 and RXFP2. Supernatants containing the viral particles were collected and filtered through a 0.45 µm filter (Corning 431220) before concentrated with the Lenti-X Concentrator (Takara 631231). Sub-confluent NCI-H295R cells were then transduced with each of the viral supernatants before limiting dilution was used to seed single cell clones into 96-well plates. Each clone was visually inspected and expanded into 24-well plates before cryopreservation and qPCR screening. The same approach was used to generate stable HEK293 lines expressing human and rat RXFP1 and RXFP2 cDNA. The cAMP assay was conducted using the Eurofin Hithunter kit per manufacturer’s instructions in a 384-well format. All dose curve fitting and EC_50_ and IC_50_ determinations were performed in Prism (v9.3.0) using the non-linear fit, variable slope (four parameter) function.

### Adrenal cell steroidogenesis assays

Cells were prepared as described above and used for assays. 55,000 cells were detached and seeded into each well of a 96-well plate. Replica plates at 3.33× final concentration of the test compound were made and cells were treated with test compounds or mAbs for 1 h before human INSL3 at approximately EC_80_ (270 pM) was added to the wells. The final test compound or mAb concentrations ranged from 10 mM to 15 nM (in 1% DMSO or PBS). Cells and the conditioned supernatant were collected at 48 h post treatment.

Total RNA was prepared using the MagMax MirVana Total RNA isolation kit (Applied Biosystems, A27828), and cDNA synthesis was performed per manufacturer’s protocol. Using 25 ng cDNA per reaction, qPCR was performed in technical duplicates. Probes for CYP11B1 (Hs01596404_m1), CYP11B2 (Hs01597732_m1), and GAPDH (Hs02786624_g1) were analyzed. RT-qPCR data was analyzed using the ΔΔCt method. ΔCt was calculated by subtracting internal GAPDH control Ct from the gene Ct. ΔΔCt was calculated by subtracting the average ΔCt of all negative control samples. Gene expression fold change (FC) was calculated as FC = 2^(−ΔΔCt)^. Dose curve fitting was performed in Prism (v9.3.0) using the variable slope (four parameter) function. Data entered as XY table, with x = [Inhibitor] (nM) and y = CYP11B2 or CYP11B1 fold change. The data was fitted with top concentration excluded, as we observed a reduction at the top vehicle (DMSO) dose (1%). The data was fitted by constraining the bottom equal to the bottom of the corresponding INSL3 dose curve fit for the set. Individual dose point data was normalized to the corresponding DMSO dose point for the corresponding DMSO control.

### Adrenal cell corticosteroid hormone secretion assays

The day after seeding, H295R cells were treated and incubated for 48 h before supernatants and cell lysates were collected. Treatments included INSL3, IgG, 1F2, and 4G6 at 0.035 pM–3.5 µM, 2D4 at 0.01 nM–1 uM, or phosphate buffered saline control. In RXFP2 antagonist assays, 3.5 nM INSL3 was used as the stimulus. Cortisol present in the adrenal cell supernatant at 48 h post treatment was detected by ELISA (R&D KGE008B). Sample dilution was optimized at 1/90 using a standard curve. Aldosterone was below the limit of detection by ELISA (R&D KGE016) and required liquid–liquid extraction (LLE) twice using methyl tetra butyl ether. Post extraction samples were dried (SPE Nitrogen dryer) and reconstituted for ELISA analysis. All samples were run in duplicates. Dose curve fitting and EC_50_ and IC_50_ determination were performed in Prism (v9.3.0 or v9.5.1) using the non-linear fit, variable slope (four parameter) function.

### DNA preparation for immunization

Rat (Q5ECL0; 20-737) and human (Q8WXD0; 20-754) RXFP2 genes were cloned into a pCAGGS plasmid (Genscript). Both constructs were designed with c-terminal T-cell epitope containing sequences from tetanus toxin (TT). The TT p30 peptide (FNNFTVSFWLRVPKVSASHLEQY) and p2 peptide (QYIKANSKFIGITE) were included in tandem to help break immune tolerance as previously described ^42,43^.

### Animals

Rats and mice used in this study were maintained in a facility adhering to AAALAC international guidelines. All experiments were approved and performed in compliance with the Institutional Animal Care and Use Committee at 23andMe or GSK and complied with the ARRIVE guidelines.

Mice (Balb/c and Swiss-webster, Jackson Laboratory) were 2–3 months old when they were used in the RXFP2 antibody campaign. For generation of anti-RXFP2 antibodies, mice were administered a priming dose of 10 µg mFlt3L, followed by weekly dosing with 2.5 µg mGM-CSF, 25 mg human full-length RXFP2 DNA, and 25 mg rat full-length RXFP2 DNA (HTV) and boosted (6 doses of hRXFP2 DNA, 15 doses of rRXFP2 DNA) based on titers from test bleeds. All doses were prepared and administered in the following manner: total DNA was prepared in lactated Ringer’s solution volume equivalent to 10% mouse body weight and delivered via hydrodynamic tail vein injections administered over 4–8 s. Serum titers were evaluated for binding in HEK293 cells stably expressing hRXFP2, rRXFP2, and parentals using flow cytometry. Three days after the final boost with either 25 µg human LDLa-linker-ratFc and 25 µg rat LDLa-linker-ratFc or 1e^6^ irradiated rRXFP2 stable cells (Rad Source RS-2000), mice were euthanized and spleens and lymph nodes were harvested. All mice maintained health throughout the study. Euthanasia was performed following the American Veterinary Medical Association guidelines. Mice were euthanized via carbon dioxide inhalation (CO_2_), using 100% CO_2_ gas at an air displacement rate of 30–70% per minute, followed by cervical dislocation.

For the pharmacokinetic study of RXFP2 mAbs in rat, 1G1, 2D4, 4B1, 4G6, 1F2, and 3F3 were prepared in phosphate buffered saline (0.100 mg/mL) with low endotoxin (≤ 0.76 EU/mL). Adult male Wistar Han rats (n = 3/mAb) were administered 1 mg/kg subcutaneously (10 mL/kg). Blood samples (100 µL) were collected in EDTA containing tubes at 0.25, 3, 6 24, 48, 96, 168 (day 7), 336 (day 14), 504 (day 21), and 672 (day 28) hours post dose, immediately centrifuged (6100 rpm), and 50 µL plasma stored at − 80 °C until assay. Rats maintained health throughout the study and were anesthetized (isoflurane to effect) and euthanized on day 28.

### Determination of RXFP2 mAb PK

Frozen rat plasma samples were thawed and analyzed using a qualified analytical method based on sample dilution, followed by Electrochemiluminescence immunoassay analysis. The lower limit of quantification (LLQ) was 0.1 µg/mL using a 10 µL aliquot of 51-fold diluted rat plasma with a higher limit of quantification (HLQ) of 10 µg/mL. RXFP2 mAbs were captured with LDLa protein and detected with sulfo-tag labeled mouse anti-rat IgG2b. The computer systems that were used to acquire and quantify data included Gyrolab Workstation Version 8.1.5 and Watson LIMS version 7.6.1. Rat pharmacokinetic parameters were calculated by non-compartmental analysis (NCA) analysis using WinNonlin Phoenix 64 (version 8.3). Values are reported in mean ± standard deviation.

### Synthesis of INSL3

Human INSL3 was purchased from Phoenix Pharmaceuticals, Inc. (Cat. No. 035-27) with a purity of 95.8%. Rat INSL3 was synthesized by Phoenix Pharmaceuticals, Inc. (A chain: SVATNAVHRCCLTGCTQQDLLGLCPH, B Chain: LRSPQPPEARAKLCGHHLVRALVRVCGGPRWSPEA. Cys-Cys (A1-A3, A2-B1 and A4-B2)) with a purity > 95%.

## Supplementary Information

Supplementary Information.

## Data Availability

The de-identified GWAS summary statistics for the 23andMe data set may be requested through 23andMe's Publication Dataset Access Program. Please visit https://research.23andme.com/collaborate/#dataset-access/ for more information and to apply to access the data. For replications, the variant-level data for the 23andMe replication dataset are fully disclosed in the manuscript. Individual-level data are not publicly available due to participant confidentiality and in accordance with the IRB-approved protocol under which the study was conducted. Individual level data from UK Biobank is available through a procedure described at www.ukbiobank.ac.uk/using-the-resource/. PheWAS results (from UK Biobank and FinnGen) and the GTEx eQTL data are publicly available as listed in the methods and results sections.
