# Author Correction: Discovery of RXFP2 genetic association in resistant hypertensive men and RXFP2 antagonists for the treatment of resistant hypertension

**DOI:** 10.1038/s41598-025-91682-w

**Published:** 2025-03-04

**Authors:** Shan-Shan Zhang, Lance Larrabee, Andrew H. Chang, Sapna Desai, Lisa Sloan, Xin Wang, Yixuan Wu, Nazia Parvez, Karen Amaratunga, Allison C. Hartman, Abby Whitnall, Joseph Mason, Nicholas P. Barton, Audrey Y. Chu, Jonathan M. Davitte, Adam J. Csakai, Caitlin Vestal Tibbetts, Audrey E. Tolbert, Heather O’Keefe, Jessie Polanco, Joseph Foley, Casey Kmett, Jonathan Kehler, Gabriela Kozejova, Feng Wang, Andrew P. Mayer, Patrick Koenig, Davide Foletti, Steven J. Pitts, Christine G. Schnackenberg

**Affiliations:** 1 Therapeutics Division, 23andMe, 349 Oyster Point Blvd, South San Francisco, CA 94080 USA; 2 Medicinal Science and Technology, GSK, Medicines Research Centre, Gunnels Wood Road, Stevenage, SG1 2NY UK; 3 Research, 23andMe, 223 N Mathilda Ave., Sunnyvale, CA 94086 USA; 4 Medicinal Science and Technology, GSK, 1250 S. Collegeville Rd., Collegeville, PA 19426 USA; 5 Genomic Sciences, GSK, 300 Technology Square, Cambridge, MA 02139 USA; 6 Medicinal Science and Technology, GSK, 200 Cambridgepark Drive, Cambridge, MA 02140 USA; 7 Novel Human Genetics Research Unit, GSK, 1250 S. Collegeville Rd., Collegeville, PA 19426 USA; 8 DMPK, GSK, 1250 S. Collegeville Rd, Collegeville, PA 19426 USA; 9 Bioanalysis, Immunogenicity and Biomarkers, GSK, 1250 S. Collegeville Rd., Collegeville, PA 19426 USA

Correction to: *Scientifc Reports* 10.1038/s41598-024-62804-7, published online 08 June 2024

The original version of this Article contained errors.

In Figure 5a, the CYP11B1 mRNA graph was a duplication of the CYP11B2 mRNA graph.

The original Figure 5 and accompanying legend appears below.

**Fig. 5:**
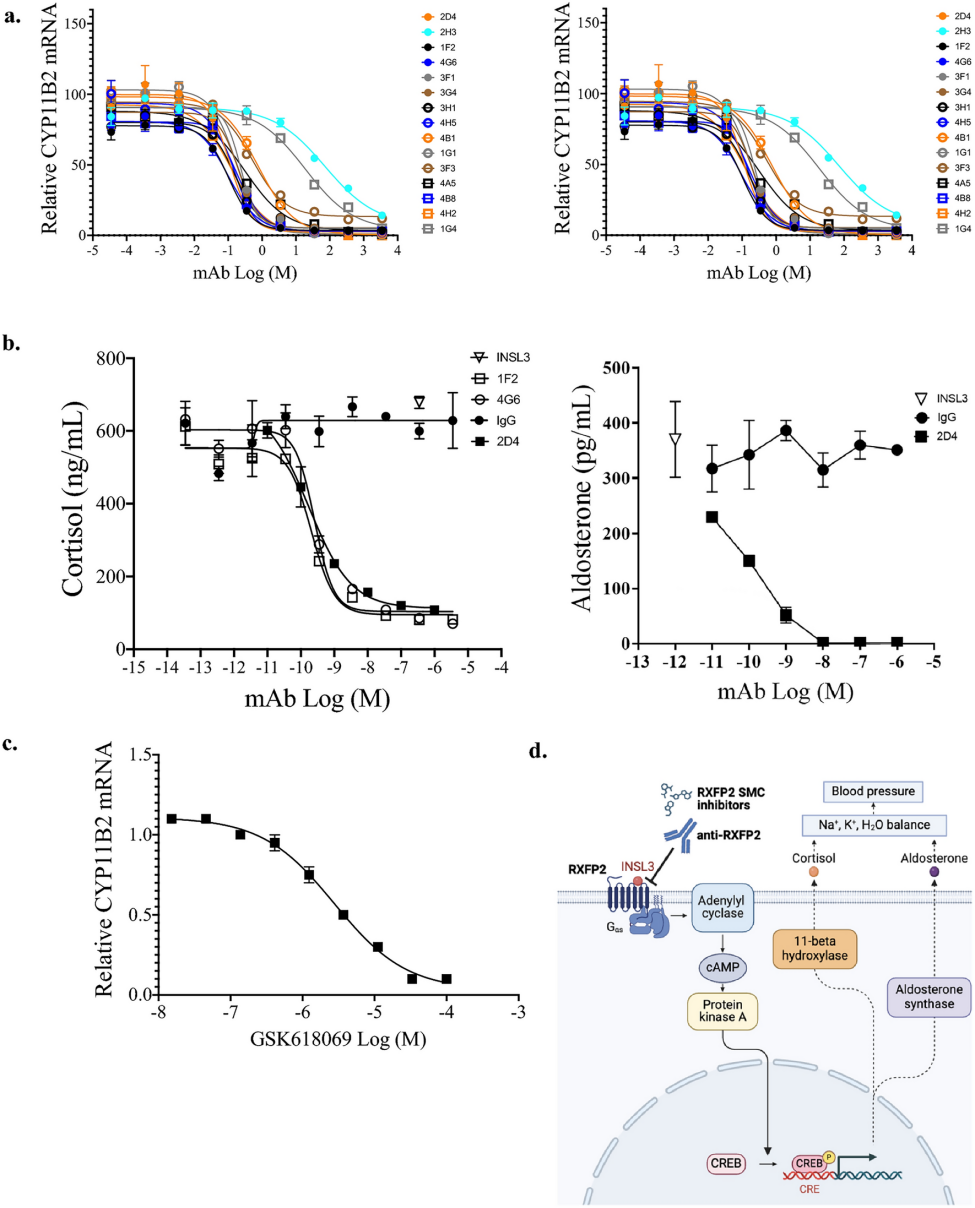
RXFP2 antagonists block INSL3-stimulated steroidogenesis and corticosteroid secretion in human adrenal cortex cells H295R stably expressing RXFP2. (**a**) Dose–response curves of RXFP2 mAb treatment in H295R cells stably expressing rat RXFP2 stimulated with 3.5 nM rat INSL3 (EC_80_) on CYP11B1 (steroid 11β-hydroxylase enzyme) and CYP11B2 (aldosterone synthase) mRNA expression levels (qPCR, relative to GAPDH control). IgG n = 6; all others n = 2. (**b**) The supernatants of cells stimulated with INSL3 and treated with the RXFP2 mAbs 1F2, 4G6, and 2D4 were assayed for cortisol (n = 2) and 2D4 was also evaluated for aldosterone (n = 2) concentration (ELISA). (**c**) Dose–response curve of RXFP2 small molecule antagonist GSK618069 in H295R cells stably expressing rat RXFP2 and stimulated with 3.5 nM rat INSL3 (EC_80_) on CYP11B2 (aldosterone synthase) mRNA expression levels (qPCR, relative to GAPDH). (**d**) Proposed hypothesis for a role of RXFP2 in causing hypertension through adrenal steroidogenesis and secretion. Graphic was created using BioRender.com. Small molecule compound (SMC).

In addition, in the Methods section, under the subheading ‘AlphaFold multimer prediction’, an incorrect reference was cited.

Consequently,

“Previous work^50^ has shown accurate antibody:antigen complex predictions are possible and an interface predicted modeling score (iPTM) score threshold of 0.75 corresponds to a possible high confidence model cutoff.”

now reads:

“Previous work^57^ has shown accurate antibody:antigen complex predictions are possible and an interface predicted modeling score (iPTM) score threshold of 0.75 corresponds to a possible high confidence model cutoff.”

The original Article has been corrected.

