# Supplementary material for: Discovery of RXFP2 genetic association in resistant hypertensive men and RXFP2 antagonists for the treatment of resistant hypertension
Source: Sci Rep. 2024 Jun 8;14:13209. doi: 10.1038/s41598-024-62804-7 (PMC11162469; doi:10.1038/s41598-024-62804-7)
Supplement: Supplementary file 1 — Supplementary Information. [file 41598_2024_62804_MOESM1_ESM.docx]

**Supplementary Information**

## Discovery of RXFP2 association in resistant hypertensive men and RXFP2 antagonists for the treatment of resistant hypertension

**Authors:** Shan-Shan Zhang^1#^, Lance Larrabee^1#^, Andrew H. Chang^1^, Sapna Desai^2^, Lisa Sloan^2^, Xin Wang^3^, Yixuan Wu^1^, Nazia Parvez^2^, Karen Amaratunga^2^, Allison C. Hartman^4^, Abby Whitnall^2^, Joseph Mason^2^, Nicholas P. Barton^2^, Audrey Y. Chu^5^, Jonathan M. Davitte^5^, Adam J. Csakai^6^, Caitlin Vestal Tibbetts^6^, Audrey E. Tolbert^6^, Heather O’Keefe^6^, Jessie Polanco^1^, Joseph Foley^7^, Casey Kmett^8^, Jonathan Kehler^9^, Gabriela Kozejova^2^, Feng Wang^8^, Andrew P. Mayer^9^, Patrick Koenig^1^, Davide Foletti^1^, Steven J. Pitts^3^, Christine G. Schnackenberg^7*^

**Author Affiliations**:

^1^Therapeutics Division, 23andMe, 349 Oyster Point Blvd, South San Francisco, CA 94080, USA.

^2^Medicinal Science and Technology, GSK, Medicines Research Centre, Gunnels Wood Road, Stevenage, UK, SG1 2NY.

^3^Research, 23andMe, 223 N Mathilda Ave., Sunnyvale CA 94086, USA.

^4^Medicinal Science and Technology, GSK, 1250 S. Collegeville Rd., Collegeville, PA, 19426, USA.

^5^Genomic Sciences, GSK, 300 Technology Square, Cambridge, MA 02139, USA.

^6^Medicinal Science and Technology, GSK, 200 Cambridgepark Drive, Cambridge, MA 02140, USA.

^7^Novel Human Genetics Research Unit, GSK, 1250 S. Collegeville Rd., Collegeville, PA, 19426, USA.

^8^DMPK, GSK, 1250 S. Collegeville Rd., Collegeville, PA, 19426, USA.

^9^Bioanalysis, Immunogenicity & Biomarkers, GSK, 1250 S. Collegeville Rd., Collegeville, PA, 19426, USA.

# Co-first authors

*Corresponding author: Christine.G.Schnackenberg@gsk.com

**Supplementary Figure S1.** The RXFP2 association in resistant hypertension men is replicated in the UK Biobank. a) Region plots. The lead SNP from the 23andMe resistant hypertension GWAS analysis (rs2146377) is circled in black; b) Summary of GWAS analyses in UK Biobank, sample sizes, total number of variants analyzed and genomic inflation factor calculation.

**a.**


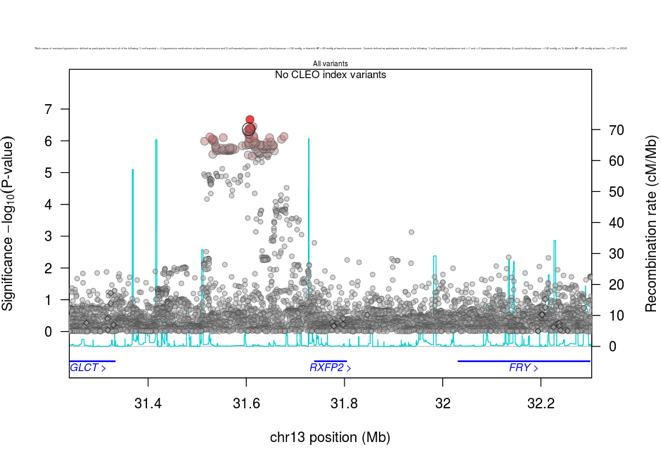


Men


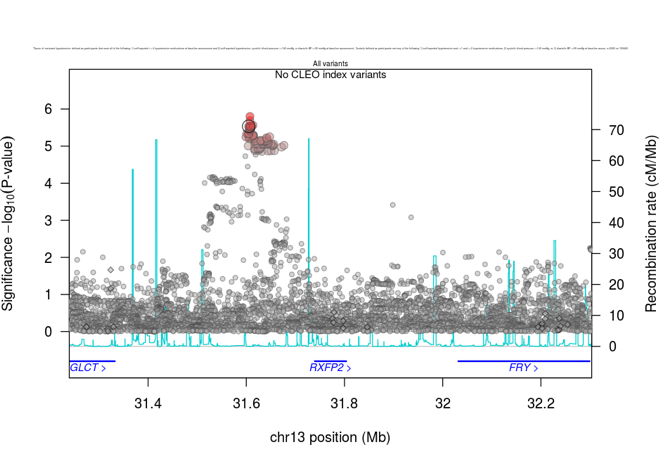


Overall


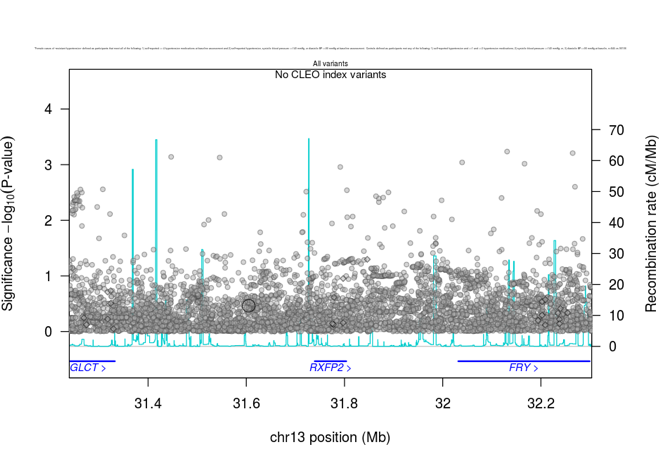


Women

**b**.

| **Analysis** | **N case** | **N control** | **N SNPs** | **Genomic inflation factor (**$\boldsymbol{\lambda}$**)** |
| --- | --- | --- | --- | --- |
| Resistant hypertension vs non-resistant hypertension (hypertension case only analysis) | 2566 | 189449 | 9353404 | 1.04 |
| Resistant hypertension vs non-resistant hypertension among women (hypertension case only analysis in women only) | 845 | 90106 | 9353686 | 1.02 |
| Resistant hypertension vs non-resistant hypertension among men (hypertension case only analysis in men only) | 1721 | 99343 | 9352979 | 1.02 |
| Resistant hypertension vs no hypertension | 2566 | 187243 | 9353193 | 1.11 |
| Resistant hypertension vs no hypertension in women only | 845 | 113409 | 9353555 | 1.05 |
| Resistant hypertension vs no hypertension in men only | 1721 | 73834 | 9351702 | 1.06 |

**Supplementary Figure S2**. Phenome-wide significant results from 23andMe for rs2146377. The effect sizes shown are with respect to the risk allele (A), with downward triangle (OR < 1) and upward triangle (OR > 1) indicating direction of effect. The dashed line indicates Bonferroni corrected p-value (p=4.5e-5), accounting for 1122 total phenotypes tested.


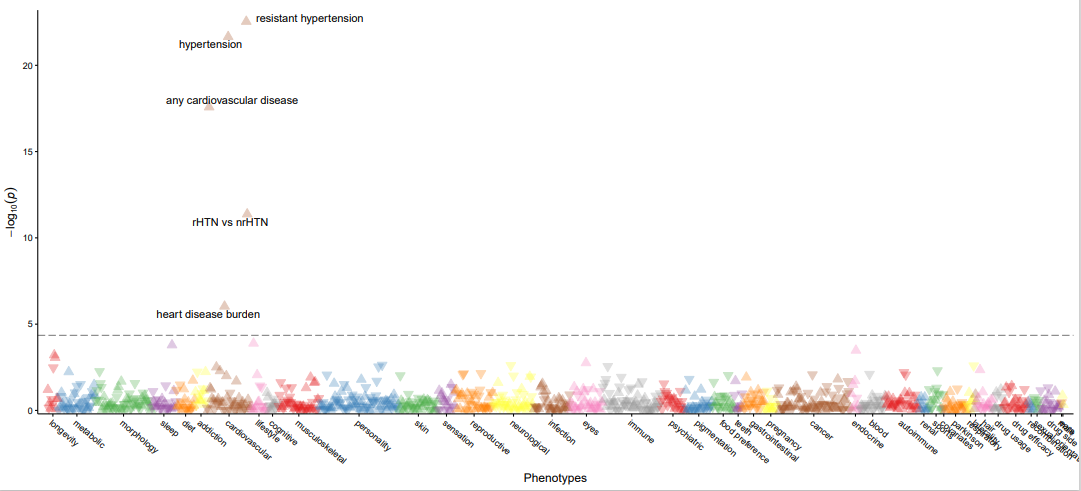


**Supplementary Figure S3**. Phenome-wide significant results for rs2146377 from publicly available UK Biobank results, <https://pheweb.org/UKB-Neale/variant/13:32179063-G-A>. The effect sizes shown are with respect to the risk allele (A), with downward triangle (OR < 1) and upward triangle (OR > 1) indicating direction of effect. The dashed line indicates Bonferroni corrected p-value (p=2.1e-5), accounting for 2416 total phenotypes tested.


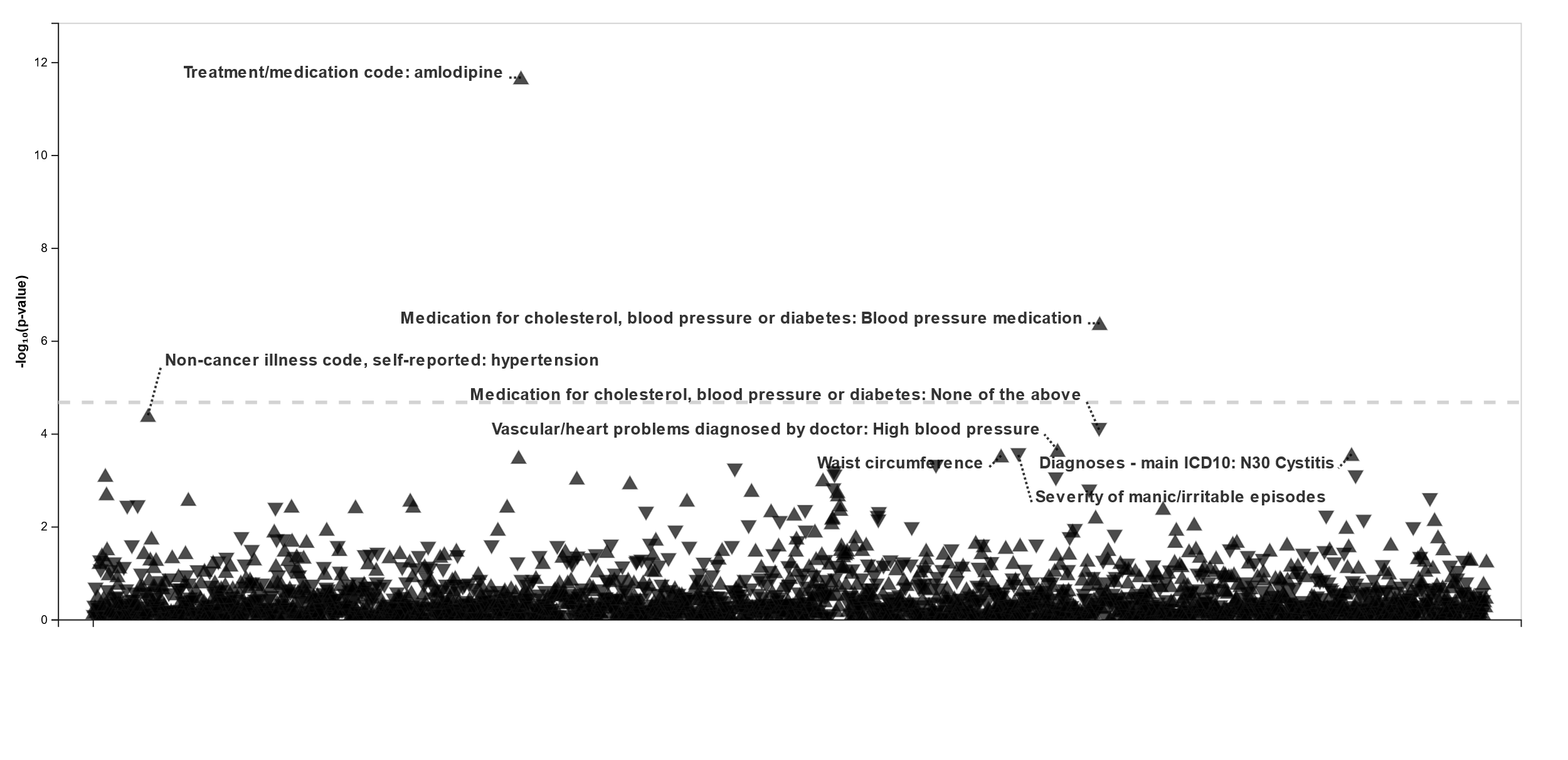


| **Phenotype** | **P-value** | **Number of samples** |
| --- | --- | --- |
| Treatment/medication code: amlodipine | 2.1e-12 | 337159 |
| [Medication for cholesterol, blood pressure or diabetes: Blood pressure medication](https://pheweb.org/UKB-Neale/pheno/6177_2) | 4.1e-7 | 154702 |

**Supplementary Figure S4**. Phenome-wide significant results for rs2146377 from publicly available FinnGen results, https://r9.finngen.fi/variant/13:31604926-G-A. The effect sizes shown are with respect to the risk allele (A), with downward triangle (OR < 1) and upward triangle (OR > 1) indicating direction of effect. The dashed line indicates Bonferroni corrected p-value (p=2.2e-5), accounting for 2272 total phenotypes and traits tested.


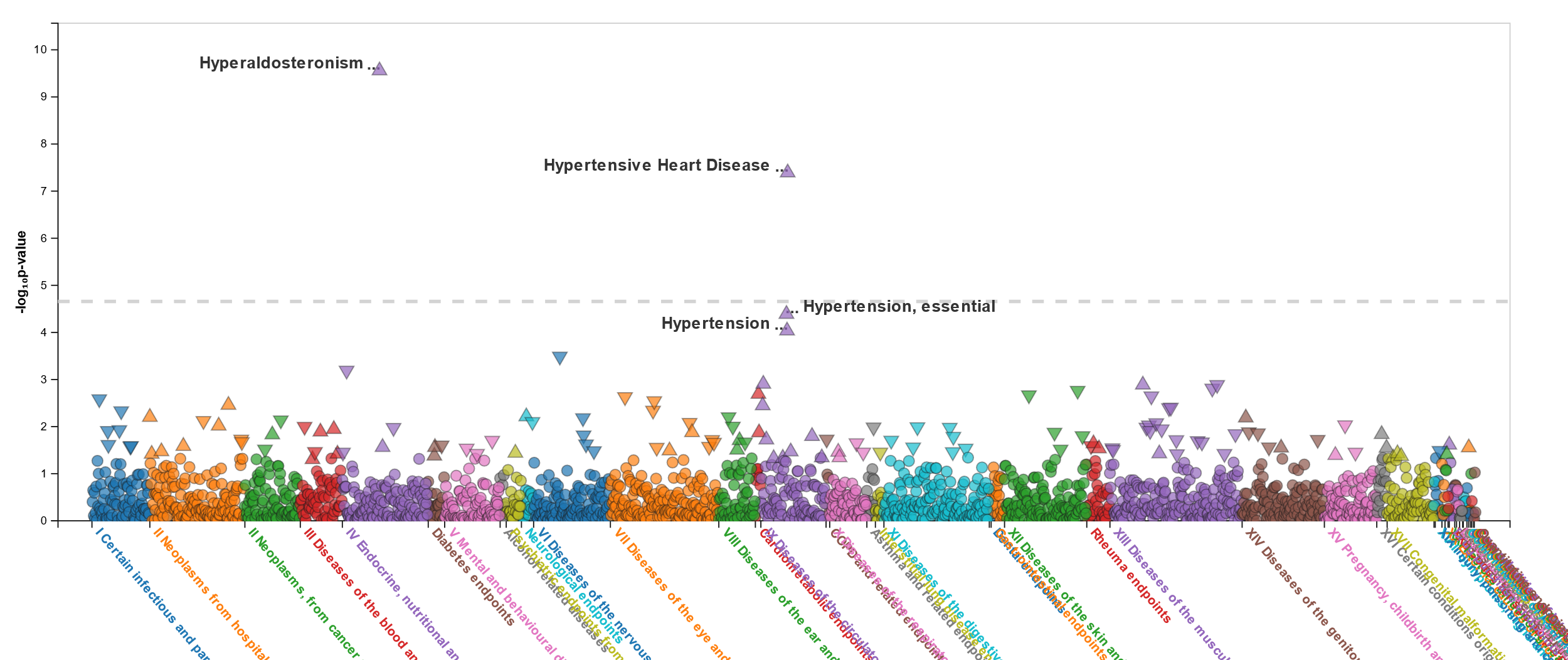


| **Phenotype** | **Odds Ratio** | **P-value** | **Number of cases** | **Number of controls** | **Allele frequency cases** | **Allele frequency controls** |
| --- | --- | --- | --- | --- | --- | --- |
| [Hyperaldosteronism](https://r9.finngen.fi/pheno/E4_HYPERALDO) | 1.40 | 2.5e-10 | 690 | 361988 | 6.2e-1 | 5.3e-1 |
| [Hypertensive Heart Disease](https://r9.finngen.fi/pheno/I9_HYPTENSHD) | 1.10 | 3.6e-8 | 8387 | 265626 | 5.6e-1 | 5.3e-1 |

**Supplementary Figure S5.** Gene expression of markers for adrenal medulla (CHGB) and cortex (NOV) in men with normotension (n=6) or hypertension (n=6). Results are expressed as gene transcript relative to GAPDH mRNA expression. Individual data points and group average + SEM are shown. No statistically significant difference in CHGB (p>0.999) and NOV (p=0.31) between groups as determined by Mann-Whitney U test.

**Supplementary Figure S6.** Compound synthesis and purity determination of BRL-37274 from HTS.





To a solution of 4-amino-3,5-dichlorobenzoic acid (10 g, 48.5 mmol) in Methanol (100 mL) was added thionyl chloride (7.08 mL, 97 mmol) at 20°C under a nitrogen atmosphere. The mixture was stirred at 70°C for 1 hr. The reaction mixture was then concentrated under reduced pressure to give a residue. The crude product, methyl 4-amino-3,5-dichlorobenzoate (14 g, 31.8 mmol, 66% yield) **BRL-37274 I1** was obtained as brown solid and used without further purification.

To a solution of methyl 4-amino-3,5-dichlorobenzoate (13 g, 59.1 mmol) in THF (100 mL) was added lithium aluminium hydride (4.48 g, 118 mmol) in small portions over 15 min at 0°C. The mixture was warmed to 20°C and stirred for 1 hr. The mixture was then cooled to 0°C and carefully quenched with water (4 mL), then diluted with 15% aqueous solution of sodium hydroxide (4 mL) and water (12 mL). The mixture was filtered and washed with EtOAc (20 mL). The mother liquor was concentrated under reduced pressure to give the product (4-amino-3,5-dichlorophenyl)methanol (9.15 g, 33.4 mmol, 57 % yield) **BRL-37274 I2** that was obtained as brown solid and was used directly.

To a solution of (4-amino-3,5-dichlorophenyl)methanol (5 g, 26.0 mmol) in THF (50 mL) was added thionyl chloride (3.80 mL, 52.1 mmol) at 0°C. The mixture was warmed to 20°C and stirred for 1 hr. The reaction mixture was concentrated to a residue of 2,6-dichloro-4-(chloromethyl)aniline (5 g, 11.88 mmol, 46 % yield). **BRL-37274 I3** was obtained as a brown solid and used in the next step without further purification.

To a mixture of 2,6-dichloro-4-(chloromethyl)aniline (5 g, 23.76 mmol) in THF (50 mL) phenyl methanethiol (8.37 mL, 71.3 mmol) was added dropwise followed by addition of triethylamine (9.93 mL, 71.3 mmol) at 0°C. The mixture was stirred at 20°C for 0.5 hr. The reaction mixture was poured into saturated aqueous NaHCO_3_ (50 mL) at 20°C, extracted with EtOAc (3 x 50 mL). The combined organic layers were washed with brine (50 mL), dried over sodium sulfate, filtered, and concentrated under reduced pressure. The residue was dissolved in dichloromethane (20 mL), and 25 g of silica gel was added. The resulting mixture was concentrated to give a dry flowing solid, and then it was loaded to Biotage using a 120 g Agela flash silica gel column, eluted with 7% to 10% EtOAc in petroleum ether. The product fraction was combined and evaporated under reduced pressure to yield 4-((benzylthio)methyl)-2,6-dichloroaniline (6.86 g, 9.20 mmol, 39 % yield), **BRL-37274 I4**, as yellow solid. ^1^H NMR (400 MHz, DMSO-d6) δ ppm 7.34-7.21 (m, 5H), 7.13 (s, 2H), 5.54 (bs, 2H), 3.78 (s, 2H), 3.62 (s, 4H) additional solvent peaks and solvent (Ethyl acetate) observed in the spectra. HPLC (UV) > 81 %. LCMS (ESI^+^) Cl isotopes observed 298.1 m/z [M+H]^+^.





To a mixture of imidazolidine-2-thione (10 g, 98 mmol) in Tetrahydrofuran (100 mL) was added sodium hydride (17.62 g, 441 mmol) at 0°C and the mixture stirred for 30 min. To this was added di-tert-butyl dicarbonate (42.7 g, 196 mmol). The mixture was stirred at 25°C for 16 hr. The reaction mixture was poured into ice cold saturated aqueous NH_4_Cl solution (500 mL). The aqueous phase was extracted with EtOAc (250 mL x 3). The combined organic phase was dried over anhydrous sodium sulfate, filtered, and concentrated under reduced pressure to give a residue. The residue was dissolved in dichloromethane (20 mL), and 20 g of silica gel was added. The resulting mixture was concentrated to give a dry flowing solid, and then it was loaded to Biotage 120 g Agela flash silica gel column (Flow rate: 200 ml/min) and eluted with 0% to 70% EtOAc in petroleum ether. The product fraction was combined and evaporated under reduced pressure to yield di-tert-butyl 2-thioxoimidazolidine-1,3-dicarboxylate (20.8 g, 68.8mmol, 70.3 % yield) ) **BRL-37274 I5** as a yellow solid. ^1^H NMR (400 MHz, CDCL3) δ ppm 3.91 (s, 4H), 1.57 (s, 18H) HPLC (UV) > 95 %. LCMS (ESI^+^) 103.2 m/z [M+H-(2xBoc+H)]^+^.





To a solution of 4-((benzylthio)methyl)-2,6-dichloroaniline (500 mg, 1.677 mmol) and di-tert-butyl 2-thioxoimidazolidine-1,3-dicarboxylate (507 mg, 1.677 mmol) in Dichloromethane (DCM) (5 mL) was added triethylamine (0.701 mL, 5.03 mmol) and mercury(II) chloride (501 mg, 1.844 mmol) at 0°C. The mixture was stirred at 25°C for 16 hr. The mixture was filtered through a Celite pad, and the filtrate was diluted with water (20 mL); the aqueous phase was extracted with dichloromethane (8 mL × 3) and the organic phase was washed with brine (8 mL × 1). The combined organic layers were dried over sodium sulfate, filtered, and the filtrate was concentrated under reduced pressure to give a residue. The residue was dissolved in acetonitrile (4 mL), and the resulting solution was purified by prep-HPLC (FA) The desired HPLC fractions were combined, lyophilized to give di-tert-butyl 2-((4-((benzylthio)methyl)-2,6-dichlorophenyl)imino)imidazolidine-1,3-dicarboxylate (30 mg, 0.041 mmol, 2 % yield), **BRL-37274 I6**, as colorless oil. ^. 1^H NMR (400 MHz, CDCL3) δ ppm 7.35-7.29 (m, 5H), 7.16 (s, 2H), 3.82 (s, 4H), 3.58 (s, 2H), 3.47 (s, 2H), 1.39 (s, 18H) HPLC (UV) > 79 %. LCMS (ESI+) 466.1 m/z [M+H-Boc+H]+.





A solution of di-tert-butyl 2-((4-((benzylthio)methyl)-2,6-dichlorophenyl)imino)imidazolidine-1,3-dicarboxylate (30 mg, 0.053 mmol) was dissolved in a 4M solution of HCl in methanol (1 mL, 4.00 mmol) was stirred at 20°C for 16 hr. The solution was then concentrated to dryness and the residue was dissolved in methyl alcohol (1 mL), and the resulting solution was purified by prep-HPLC (FA modifier, the detail condition as shown above 30- 70% Acetonitrile). The HPLC fractions were combined, lyophilized to give 8.4 mg N-(4-((benzylthio)methyl)-2,6-dichlorophenyl)-4,5-dihydro-1H-imidazol-2-amine (43.3 % yield) **BRL-37274** as white solid. ^1^H NMR (400 MHz, DMSO-d6) δ ppm 8.1 (bs, ~2H), 7.46 (s, 2H), 7.33-7.26 (m, 5 H), 3.72 (s, 4H), 3.60 (s, 4H) HPLC (UV) > 95 %. LCMS (ESI^+^) Cl isotopes observed 365.9 and 367.9 m/z [M+H]^+^.

**Supplementary Figure S7.** Compound synthesis and purity determination of GSK618069 from HTS.





To a vial containing tert-butyl piperazine-1-carboxylate (1.288 g, 6.91 mmol) and potassium carbonate (1.737 g, 12.57 mmol) was added a solution of 1,3-difluoro-2-nitrobenzene (1 g, 6.29 mmol) in dimethyl sulfoxide (DMSO) (5 mL). The reaction was stirred at RT for 17 hrs. The reaction mixture was diluted with diethyl ether (150 mL) and washed with water (2 x 150 mL). The organics were dried by filtration through hydrophobic frit and concentrated to give 2.25 g of the desired crude S_N_Ar product tert-butyl 4-(3-fluoro-2-nitrophenyl)piperazine-1-carboxylate, **GSK618069 I1**.

The 2.25 g of yellow oil, **GSK618069 I1**, was dissolved in 1,4-dioxane (15 mL) and 4M HCl solution in 1,4-dioxane (8 mL, 32.0 mmol) was added. The resulting solution was left to stir at ambient temperature for 15 hrs. The reaction mixture was concentrated under reduced pressure and dried in a vacuum oven at 40°C for 3 hrs to give 1.61 g the desired crude product 1-(3-fluoro-2-nitrophenyl)piperazine **GSK618069 I2**.

To a solution of cyclopentanamine (0.579 mL, 5.87 mmol) in water (50 mL) at 0°C was added CDI (1143 mg, 7.05 mmol) while cooling with an ice bath. This solution was stirred at 0°C for 1 hr before the crude product of the previous reaction, **GSK618069 I2**, was added and the reaction mixture was stirred at ambient temperature for 16 hrs. The reaction mixture was diluted with water and extracted with ethyl acetate. The organics were dried by filtration through a hydrophobic frit and concentrated. The material was purified (CombiFlash) using a 40 g Si column (340 mL/min) eluting a 0-100% cyclohexane-EtOAc gradient with peak collection at 254/280 nm. The appropriate fractions were combined and concentrated to give N-cyclopentyl-4-(3-fluoro-2-nitrophenyl)piperazine-1-carboxamide **GSK618069 I3** (805 mg, 41%) ^1^H NMR (DMSO-d6, 400 MHz): δ (ppm) 7.58 (td, J = 8.5, 6.5 Hz, 1H), 7.26-7.17 (m, 2H), 6.28 (d, J = 6.8 Hz, 1H), 3.95-3.80 (m, 1H), 3.42-3.32 (m, 4H), 2.99-2.90 (m, 4H), 1.83-1.73 (m, 2H), 1.68-1.56 (m, 2H), 1.52-1.34 (m, 4H). LCMS (HpH) rt = 1.10 min. (ESI^+^) 337.0 [M+H]^+^ >95 % purity by UV.





In a glovebox, to a solution of tert-butyl 4-aminopiperidine-1-carboxylate (589 mg, 2.94 mmol) and N-cyclopentyl-4-(3-fluoro-2-nitrophenyl)piperazine-1-carboxamide (660 mg, 1.962 mmol) in dimethyl sulfoxide (DMSO) (20 mL) was added N-ethyl-N-isopropylpropan-2-amine (0.685 mL, 3.92 mmol). The resulting solution was heated to 100°C with stirring for 25 hrs. The reaction was allowed to cool to room temperature, then diluted with water (200 mL) and EtOAc (30 mL). The organic layer was separated, and the aqueous layer extracted with EtOAc (3 x 20 ml). The combined organics were filtered through a hydrophobic frit and reduced in vacuo to give the crude product. This sample was loaded onto celite and purified by normal phase chromatography (0-100% EtOAc in cyclohexane) using a 40 g silica cartridge over 20 CVs. The desired fractions were combined and concentrated in vacuo to give an orange solid tert-butyl 4-((3-(4-(cyclopentylcarbamoyl)piperazin-1-yl)-2-nitrophenyl)amino)piperidine-1-carboxylate **GSK618069 I4** (679 mg, 57%) used in next reaction semi-crude. LCMS (HpH) rt = 1.26 min. (ESI^+^) 517.3 [M+H]^+^ > 95% purity by UV.

A solution of tert-butyl 4-((3-(4-(cyclopentylcarbamoyl)piperazin-1-yl)-2-nitrophenyl)amino)piperidine-1-carboxylate (675 mg, 1.307 mmol) and ammonium formate (824 mg, 13.07 mmol) in methanol (10 mL) was evacuated and backfilled with nitrogen 3 times. Then the round bottomed flask was charged with palladium on carbon (10 wt%) (139 mg, 0.131 mmol) and the reaction was heated to 80°C for 1 hr. After this time the reaction mixture was filtered through celite (2.5 g) and eluted with methanol (3 column volumes). The combined organics were reduced in vacuo to give the crude product tert-butyl 4-((2-amino-3-(4-(cyclopentylcarbamoyl)piperazin-1-yl)phenyl)amino)piperidine-1- carboxylate **GSK618069 I5** (607 mg, 59%).

To a solution of tert-butyl 4-((2-amino-3-(4-(cyclopentylcarbamoyl)piperazin-1-yl)phenyl)amino)piperidine-1-carboxylate, **GSK618069 I5** , (607 mg, 1.247 mmol) in toluene (5 mL) was added isonicotinaldehyde (0.141 mL, 1.497 mmol) and sodium hydrosulfite (217 mg, 1.247 mmol). The reaction was stirred to 80°C for 2 hrs. The reaction was concentrated in vacuo, but the crude product **GSK618069 I6** was immediately resuspended in Dichloromethane (DCM) (10 mL). To this solution, was added 4 M HCl in 1,4-dioxane (5 mL, 1.247 mmol) and the reaction stirred at room temperature for 1 hr. The reaction was quenched with saturated aqueous NaHCO3 and extracted with ethyl acetate. The combined organics were filtered through a hydrophobic frit and concentrated in vacuo to give the crude product. This sample was redissolved in DMSO (7 mL), and 2 mL of this solution was split equally across two mass directed auto prep (MDAP) run (formic, modifier 15-55% B). The desired fractions from the two MDAP runs were combined and concentrated in vacuo, then redissolved in 1:1 DCM/MeOH to transfer to a vial, then blown down at 40°C for 16 hr under a stream of nitrogen to give the yellow solid N-cyclopentyl-4-(1-(piperidin-4-yl)-2-(pyridin-4-yl)-1H-benzo[d]imidazol-4-yl)piperazine-1-carboxamide as the formic acid salt **GSK618069** (37 mg, 5%, >95% pure) ^1^H NMR (DMSO-d6, 400 MHz): δ (ppm) 8.80 (d, J = 5.9 Hz, 2H), 8.28 (s, 1H), 7.69 (d, J = 5.9 Hz, 2H), 7.51 (br d, J = 7.9 Hz, 1H), 7.19 (br t, J = 7.9 Hz, 1H), 6.65 (d, J = 7.9 Hz, 1H), 6.28 (d, J = 6.9 Hz, 1H), 4.47-4.66 (m, 1H), 3.83-4.02 (m, 1H), 3.31 (br s, 2H), 2.81-3.04 (m, 2H), 2.65 (br q, J = 11.8 Hz, 2H), 2.02 (br d, J = 10.8 Hz, 2H), 1.74-1.85 (m, 2H), 1.56-1.71 (m, 2H), 1.33-1.56 (m, 4H) + broad lump 2ppm - 4ppm (assumed 8H, piperazine). LCMS (HpH). rt = 0.81 min. (ESI^+^) 474.3 [M+H]^+^ >95% purity by UV.

**Supplementary Figure S8.** Compound synthesis and purity determination of GSK4761528 from ELT screen.

**N-methyl-4-(1-methyl-4-(((1-methyl-1H-imidazol-4-yl)methyl)amino)-6-oxo-1,6-dihydropyridazin-3-yl)benzamide:** To a solution of 5,6-dichloro-2-methylpyridazin-3(2H)-one (59 mg, 0.33 mmol) in EtOH (1 mL) was added (1-methyl-1H-imidazol-4-yl)methanamine (128 mg, 1.15 mmol) and DIPEA (0.23 mL, 1.32 mmol). The reaction was heated at 130 °C in a microwave reactor for 25 minutes. LCMS showed full conversion to the desired S_N_Ar product (6-chloro-2-methyl-5-(((1-methyl-1H-imidazol-4-yl)methyl)amino)pyridazin-3(2H)-one). The mixture was concentrated to dryness, dissolved in DCM (10 mL), washed with water (10 mL), saturated NaCl (10 mL), dried over Na_2_SO_4_, filtered, and concentrated to dryness. The residue obtained was dissolved in DMF (1 mL). To the solution was added (4-(methylcarbamoyl)phenyl)boronic acid (56 mg, 0.32 mmol), K_3_PO_4_ (0.631 mL, 0.631 mmol, 1 M), and XPhos Pd G2 (19 mg, 0.024 mmol). The resulting mixture was degassed under vacuum and backfilled with nitrogen (3x), and then heated overnight at 80 °C. LCMS showed conversion to the desired Suzuki product. The mixture was purified via preperative HPLC: gradient of 2% to 30% B (mobile phase A = 0.1% formic acid in water; mobile phase B = 0.1% formic acid in acetonitrile). Obtained N-methyl-4-(1-methyl-4-(((1-methyl-1H-imidazol-4-yl)methyl)amino)-6-oxo-1,6-dihydropyridazin-3-yl)benzamide (6.3 mg, 10%). ^1^H NMR (400 MHz, MeCN-*d*_3_) δ ppm 7.48 (d, *J*=1.0 Hz, 2H), 7.20 (d, *J*=1.0 Hz, 2H), 6.89 - 7.02 (m, 1H), 6.62 - 6.78 (m, 1H), 6.49 (s, 1H), 5.38 (s, 1H), 4.77 - 4.98 (m, 1H), 3.67 - 3.77 (m, 2H), 3.20 - 3.22 (m, 3H), 3.20 - 3.20 (m, 2H), 2.46 - 2.54 (m, 3H), 1.55 - 1.59 (m, 3H), 1.54 - 1.55 (m, 1H). HPLC (UV) > 95 %. LCMS (ESI^+^) 353.19 m/z [M+H]^+^.

**Supplementary Figure S9.** Compound synthesis and purity determination of GSK4720639 from ELT screen.

**3-(((2-(dimethylamino)-9-methyl-9H-purin-8-yl)thio)methyl)-6-fluoro-1-methylquinoxalin-2(1H)-one:** To a solution of 2,4-dichloro-5-nitropyrimidine (243 mg, 1.25 mmol) and DIPEA (0.656 mL, 3.76 mmol) in EtOH (2 mL) was added methanamine (0.626 mL, 1.25, 2 M in THF). LCMS after 10 minutes indicates full conversion to the desired S_N_Ar product (2-chloro-N-methyl-5-nitropyrimidin-4-amine). The reaction mixture was concentrated to dryness. The residue obtained was dissolved in EtOH (2 mL). DIPEA (0.463 mL, 2.65 mmol), and dimethylamine (0.530, 1.06 mmol, 2 M in THF) were added to the solution and the reaction was allowed to stir overnight. LCMS shows formation of the desired S_N_Ar product (N^2^,N^2^,N^4^-trimethyl-5-nitropyrimidine-2,4-diamine). The reaction was concentrated to dryness. The residue obtained was dissolved in DMF (1.5 mL). Palladium on carbon (108 mg, 0.101 mmol, 10 wt%) was added to the solution. The resulting mixture was degassed under vacuum and backfilled with nitrogen (3x). The resulting mixture was degassed under vacuum and backfilled with hydrogen (3x). The mixture was allowed to stir under hydrogen (1 atm, balloon) overnight. After this time, LCMS indicates formation of the desired N^2^,N^2^,N^4^-trimethylpyrimidine-2,4,5-triamine. The mixture was degassed under vacuum and backfilled with nitrogen (3x). The mixture was filtered through a plug of Celite (rinsing with 0.5 mL of DMF). To the filtrate was added 1,1′-thiocarbonyldiimidazole (90 mg, 0.51 mmol). The resulting mixture was allowed to stir for 3 hours. LCMS indicates formation of the desired 2-(dimethylamino)-9-methyl-9H-purine-8-thiol. Solid K_2_CO_3_ (83 mg, 0.60 mmol) and 3-(bromomethyl)-6-fluoro-1-methylquinoxalin-2(1H)-one (65 mg, 0.24 mmol) and the resulting mixture was allowed to stir for 30 minutes. LCMS shows formation of the desired final product. The reaction was concentrated to dryness and purified by preperative HPLC: gradient of 5% to 95% B (mobile phase A = 0.1% formic acid in water; mobile phase B = 0.1% formic acid in acetonitrile). Obtained 3-(((2-(dimethylamino)-9-methyl-9H-purin-8-yl)thio)methyl)-6-fluoro-1-methylquinoxalin-2(1H)-one (8.5 mg, 8%). ^1^H NMR (400 MHz, DMSO-*d*_6_) δ ppm 8.53 (s, 1H), 7.63 - 7.67 (m, 2H), 7.56 - 7.60 (m, 1H) 4.77 (s, 2H), 3.67 (s, 3H), 3.57 (s, 3H), 3.16 (s, 6H). HPLC (UV) > 95 %. LCMS (ESI^+^) 400.22 m/z [M+H]^+^.

**Supplementary Figure S10.** Compound synthesis and purity determination of GSK4762707 from ELT screen.

**(R)-5-(N-(1-((4-(1H-indol-5-yl)benzyl)(methyl)amino)-3-methyl-1-oxobutan-2-yl)sulfamoyl)-1-methyl-1H-pyrrole-2-carboxylic acid:** To a solution of (tert-butoxycarbonyl)-D-valine (109 mg, 0.500 mmol) and HATU (190 mg, 0.500 mmol) in DMF (2.5 mL) was added DIPEA (0.261 mL, 1.5 mmol). The resulting mixture was allowed to stir for 10 minutes. After this time, 1-(4-bromophenyl)-N-methylmethanamine (0.072 mL, 0.50 mmol) was added. The resulting mixture was allowed to stir over three nights. After this time, the reaction was concentrated to dryness. The residue obtained was filtered through a plug of silica gel, eluting with 30% EtOAc/70% Hexanes. The filtrate was concentrated to dryness, giving the desired crude material (167 mg, 84%). The crude material was taken up into methanol (4 mL), and HCl (1 mL, 4 M in dioxane), and the resulting mixture was stirred overnight. The reaction was concentrated to dryness to give crude (R)-2-amino-N-(4-bromobenzyl)-N,3-dimethylbutanamide hydrochloride (140 mg, 0.417 mmol). The solid obtained was diluted with THF (4.2 mL) and cooled to 0 °C in an ice bath. To the mixture was added methyl 5-(chlorosulfonyl)-1-methyl-1H-pyrrole-2-carboxylate (109 mg, 0.459 mmol) and DIPEA (0.291 mL, 1.67 mmol). The resulting mixture was allowed to stir at 0 °C for 30 minutes. After this time, the ice bath was removed and the reaction was allowed to stir at room temperature overnight. After this time the reaction was concentrated to dryness. The residue obtained was diluted with EtOAc (30 mL), washed with saturated NaHCO_3_ (10 mL), washed with saturated NaCl (10 mL), dried over MgSO_4_, filtered, and concentrated to dryness to give crude methyl (R)-5-(N-(1-((4-bromobenzyl)(methyl)amino)-3-methyl-1-oxobutan-2-yl)sulfamoyl)-1-methyl-1H-pyrrole-2-carboxylate (163 mg, 78%). A mixture of the crude material (50 mg, 0.103 mmol), (1H-indol-5-yl)boronic acid (20 mg, 0.12 mmol), Xphos Pd G2 (8.1 mg, 0.010 mmol), and K_3_PO_4_ (0.310 mL, 0.310 mmol, 1 M), in DMF (0.516 mL) was degassed by sparging with nitrogen gas for several minutes. The resulting mixture was heated at 80 °C overnight. After this time, the mixture was diluted with 20% methanol/80% DCM (30 mL) and was filtered through a plug of Celite. The filtrate was concentrated to dryness and purified by preperative HPLC: gradient of 40% to 95% B (mobile phase A = 0.1% formic acid in water; mobile phase B = 0.1% formic acid in acetonitrile). Obtained (R)-5-(N-(1-((4-(1H-indol-5-yl)benzyl)(methyl)amino)-3-methyl-1-oxobutan-2-yl)sulfamoyl)-1-methyl-1H-pyrrole-2-carboxylic acid (14 mg, 26%). ^1^H NMR (400 MHz, Methanol-*d*_4_, **mixture of ≈ 3:1 rotamers**) δ ppm 8.24 (s, 0.2H), 7.72 - 7.65 (m, 1.4H), 7.57 - 7.52 (m, 0.7H), 7.51 - 7.45 (m, 2H), 7.37 - 7.26 (m, 3.8H), 7.21 - 7.2 (m, 1.7H), 7.05 - 6.97 (m, 3.9H), 6.42 - 6.36 (m, 1.3H), 4.55 - 4.38 (m, 1H), 4.34 (s, 2H), 4.08 - 4.03 (m, 0.4H), 4.00 - 3.95 (m, 1H), 3.81 (s, 3H), 3.79 (s, 1H), 2.84 (s, 3H), 2.68 (s, H), 1.95 - 1.81 (m, 1.6H), 0.96 - 0.76 (m, 8.5H). HPLC (UV) > 95 %. LCMS (ESI^+^) 522.91 m/z [M+H]^+^.

**Supplementary Figure S11.** Compound synthesis and purity determination of GSK4762706 from ELT screen.

**3-(2-(3,4-dimethylphenyl)acetamido)-5-hydroxy-N-(1-(methylcarbamoyl)cyclopropyl) benzamide:** To a solution of 1-((tert-butoxycarbonyl)amino)cyclopropane-1-carboxylic acid (0.101 g, 0.500 mmol) in THF (2.5 mL) was added carbonyldiimidazole (0.162 g, 1.00 mmol). The mixture was allowed to stir for 2 hours. After this time, methanamine (1 mL, 2 mmol, 2 M in THF). The resulting mixture was allowed to stir overnight. After this time, the reaction was diluted with saturated NaHCO_3_ (5 mL), and water (5 mL). The mixture was extracted with EtOAc (3 x 10 mL) and the combined organic layers were washed with saturated NaCl (10 mL), dried over MgSO_4_, filtered, and concentrated to dryness. The crude material was further dried by with diluting with toluene (3 mL) and concentrating to dryness. The crude material was dissolved in dioxane (2.5 mL) and methanol (0.5 mL). HCl (2.5 mL, 10 mmol, 4 M) was added, and the reaction was allowed to stir for 5 hours. The reaction was concentrated to dryness to give the desired amine HCl salt. In a separate vial, a solution of 3-hydroxy-5-nitrobenzoic acid (31 mg, 0.17 mmol), HATU (64 mg, 0.17 mmol), and DIPEA (0.177 mL, 1.02 mmol) in DMF (0.846 mL) was stirred for 5 minutes. After this time, 1-amino-N-methylcyclopropane-1-carboxamide hydrochloride (31 mg, 0.20 mmol, from the original reaction vial) was added. The resulting mixture was allowed to stir overnight. After this time, the reaction was concentrated to dryness. The residue obtained was diluted with EtOAc (30 mL), washed with 5% LiCl (2 x 10 mL), washed with saturated NaCl (10 mL), dried over MgSO_4_, filtered, and concentrated to dryness to give crude 3-hydroxy-N-(1-(methylcarbamoyl)cyclopropyl)-5-nitrobenzamide. A portion of the crude material (23 mg, 0.082 mmol) and palladium on carbon (8.8 mg, 0.0082 mmol, 10 wt%) was added to the solution. The resulting mixture was degassed under vacuum and backfilled with nitrogen (3x). The resulting mixture was degassed under vacuum and backfilled with hydrogen (3x). The mixture was allowed to stir under hydrogen (1 atm, balloon) for 90 minutes. After this time, the reaction was degassed under vacuum and backfilled with nitrogen. The mixture was filtered through Celite, rinsing with 10% methanol/90% DCM. The filtrate was concentrated to dryness to give crude 3-amino-5-hydroxy-N-(1-(methylcarbamoyl)cyclopropyl)benzamide. In a separate vial, a mixture of 2-(3,4-dimethylphenyl)acetic acid (13 mg, 0.080 mmol), HATU (31 mg, 0.08 mmol), and DIPEA (0.056 mL, 0.32 mmol) in DMF (0.4 mL) was allowed to stir for 5 minutes. After this time, 3-amino-5-hydroxy-N-(1-(methylcarbamoyl)cyclopropyl)benzamide (20 mg, 0.080 mmol) was added and the reaction was allowed to stir overnight. The reaction was filtered and purified by preperative HPLC: gradient of 5% to 58% B (mobile phase A = 0.1% formic acid in water; mobile phase B = 0.1% formic acid in acetonitrile). Obtained 3-(2-(3,4-dimethylphenyl)acetamido)-5-hydroxy-N-(1-(methylcarbamoyl)cyclopropyl)benzamide (3 mg, 9%). ^1^H NMR (400 MHz, Methanol-*d*_4_,) δ ppm 7.46 - 7.42 (m, 1H), 7.27 - 7.23 (m, 1H), 7.12 (br s, 1H), 7.10 - 7.04 (m, 2H), 7.04 - 7.00 (m, 1H), 3.59 (s, 2H), 2.73 (s, 3H), 2.26 (s, 3H), 2.24 (s, 3H), 1.55 - 1.49 (m, 2H), 1.12 - 1.05 (2H). HPLC (UV) > 95 %. LCMS (ESI^+^) 396.08 m/z [M+H]^+^.

**Supplementary Figure S12.** a) The amino acid sequences for human, rat and mouse RXFP2 with indications for the LDLa, Linker and LRR domains. b) The Alphafold prediction models of rat LDLa-linker and LRR domains.

**a.**


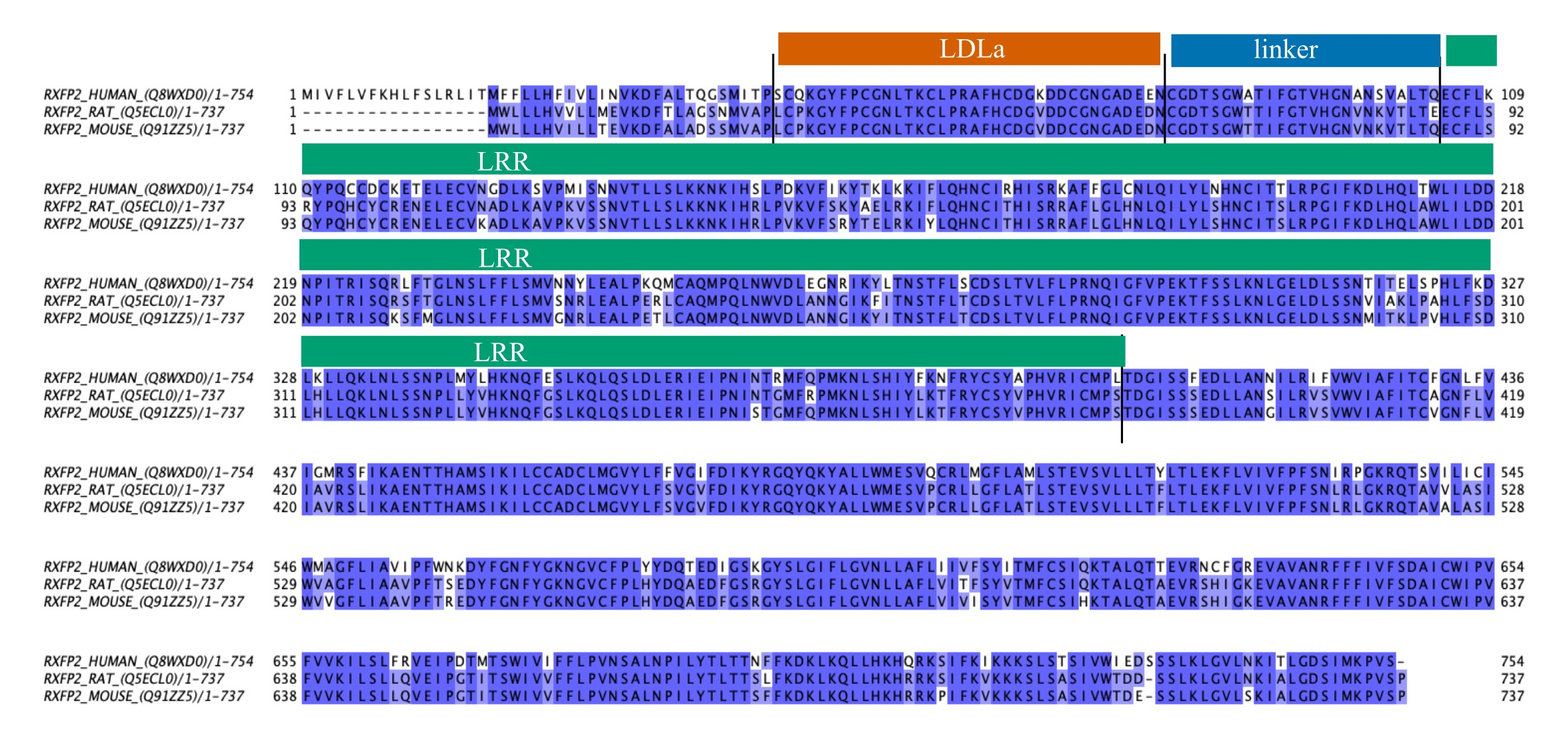


**b.**


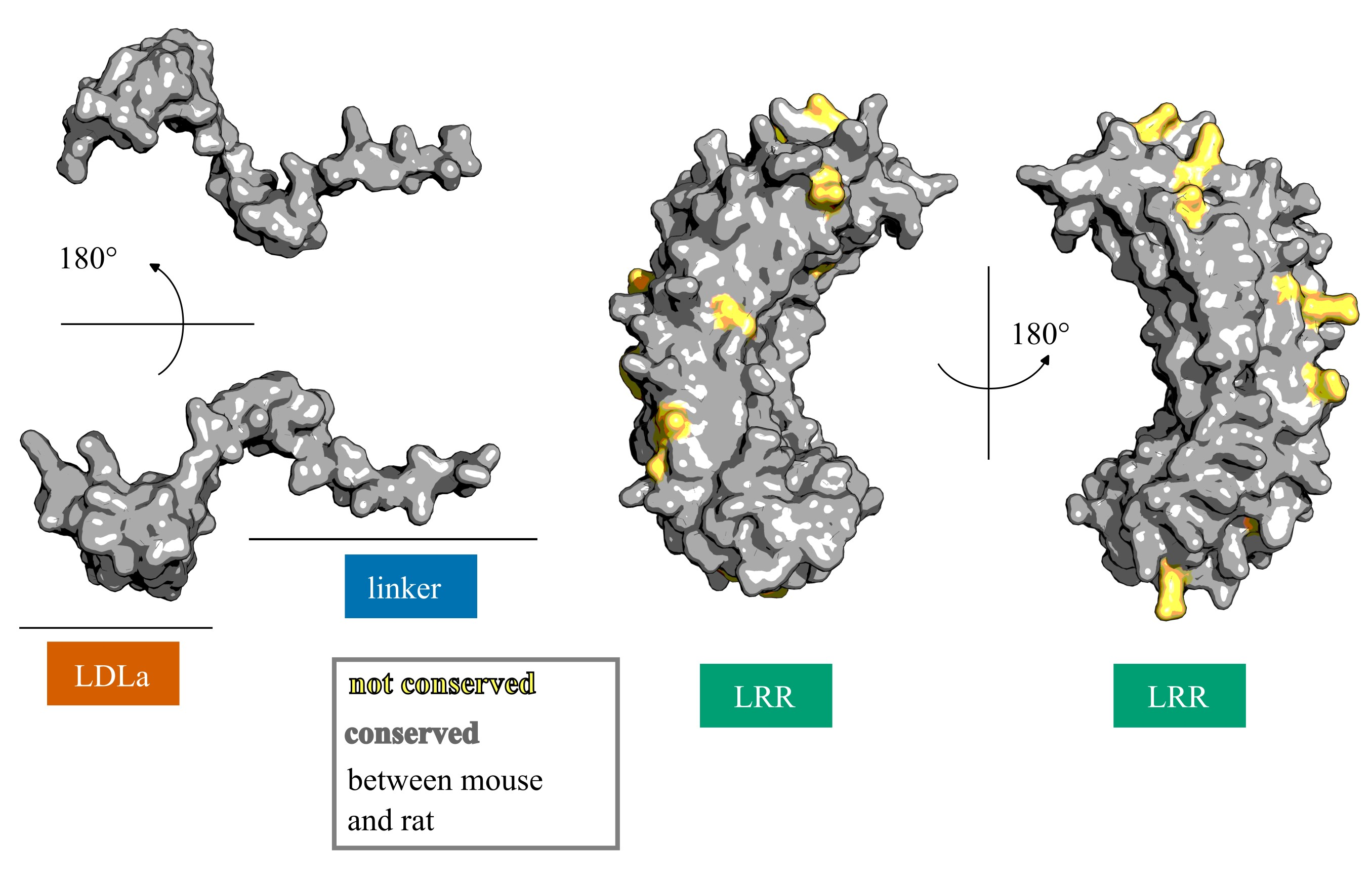


**Supplementary Figure S13.** RXFP2 mAbs epitope characterization. a) RXFP2 mAbs distribution and binning. b) Human/rat cross reactive RXFP2 mAbs binding in a peptide ELISA.

**a**.


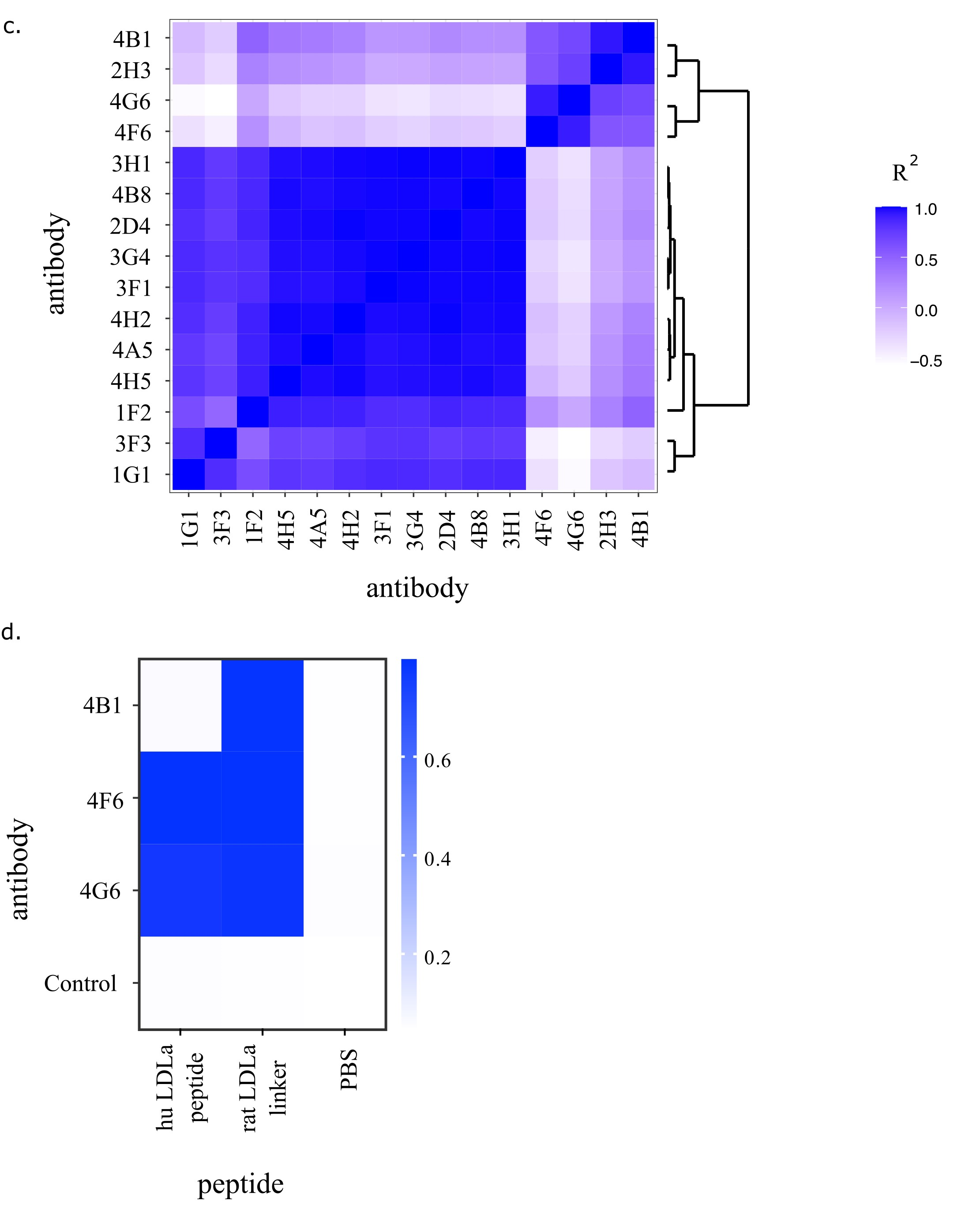


**b.**

**Supplementary Figure S14.** Characterization of RXFP screening and profiling assays for the RXFP2 mAb discovery campaign. RXFP2 ligands stimulate cAMP generation in HEK293 cells with stable expression of a RXFP receptor. a) Treatment with INSL3 for 30 minutes dose-dependently increases cAMP in HEK293 cells stably expressing human RXFP2 (EC_50_ = 2.9 nM, n=3). b) Treatment with INSL3 for 30 minutes dose-dependently increases cAMP in HEK293 cells stably expressing rat RXFP2 (EC_50_ = 8.4 nM, n=3). c) Treatment with relaxin (RLN2) for 30 minutes dose-dependently increases cAMP in HEK293 cells stably expressing rat RXFP1 (EC_50_ = 29.5 nM, n=3).

**a. HEK-hRXFP2**

**b. HEK-rRXFP2**

**c. HEK-rRXFP1**

**Supplementary Figure S15.** AlphaFold multimer prediction of a) 4B1 and b) 4G6.

**a.**


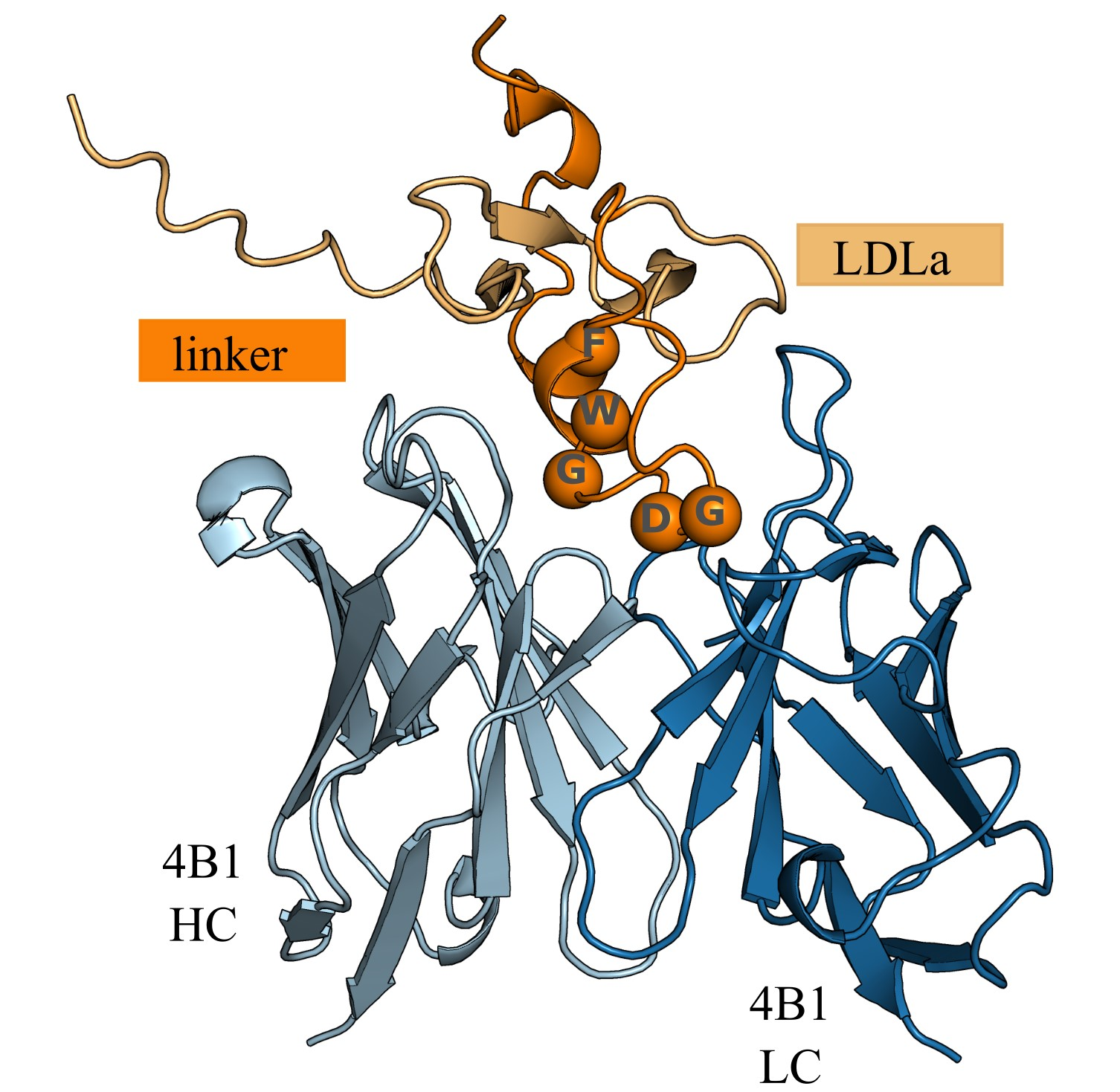


**b.**


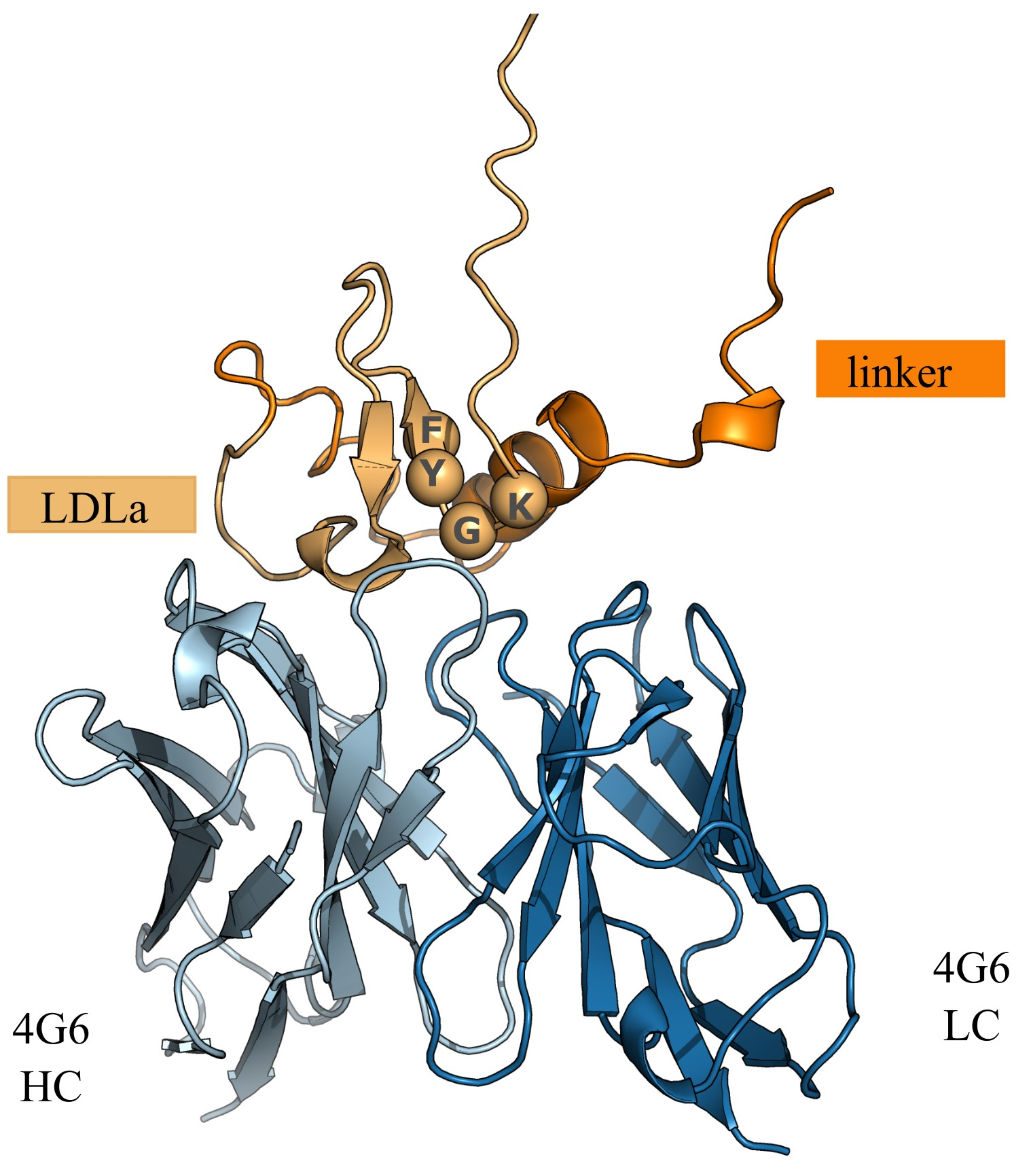


**Supplementary Figure S16.** Sequences of the RXFP2 LDLa-linker and extracellular domain (ECD) recombinant proteins that were generated for use in the RXFP2 mAb campaign.

Rat RXFP2-LDLa-linker(20-88)-rat Fc

GSNMVAPLCPKGYFPCGNLTKCLPRAFHCDGVDDCGNGADEDNCGDTSGWTTIFGTVHGNVNKVTLTEEASGGGDDDDKGGGSVPEAAGGPSVFIFPPKPKDILLISQNAKVTCVVVDVSEEEPDVQFSWFVNNVEVHTAQTQPREEQYNSTFRVVSALPIQHQDWMSGKEFKCKVNNKALGSPIEKTISKPKGLVRKPQVYVMGPPTEQLTEQTVSLTCLTSGFLPNDIGVEWTSNGHIEKNYKNTEPVMDSDGSFFMYSKLNVERSRWDSRAPFVCSVVHEGLHNHHVEKSISRPPGGGGGS

Human RXFP2-LDLa-linker (38-105)-rat Fc

GSMITPSCQKGYFPCGNLTKCLPRAFHCDGKDDCGNGADEENCGDTSGWATIFGTVHGNANSVALTQEASGGGDDDDKGGGSVPEAAGGPSVFIFPPKPKDILLISQNAKVTCVVVDVSEEEPDVQFSWFVNNVEVHTAQTQPREEQYNSTFRVVSALPIQHQDWMSGKEFKCKVNNKALGSPIEKTISKPKGLVRKPQVYVMGPPTEQLTEQTVSLTCLTSGFLPNDIGVEWTSNGHIEKNYKNTEPVMDSDGSFFMYSKLNVERSRWDSRAPFVCSVVHEGLHNHHVEKSISRPPGGGGGS

Rat Fc negative control

GGGSVPEAAGGPSVFIFPPKPKDILLISQNAKVTCVVVDVSEEEPDVQFSWFVNNVEVHTAQTQPREEQYNSTFRVVSALPIQHQDWMSGKEFKCKVNNKALGSPIEKTISKPKGLVRKPQVYVMGPPTEQLTEQTVSLTCLTSGFLPNDIGVEWTSNGHIEKNYKNTEPVMDSDGSFFMYSKLNVERSRWDSRAPFVCSVVHEGLHNHHVEKSISRPPGGGGGS

Human RXFP2 ECD (238-360)

SMVNNYLEALPKQMCAQMPQLNWVDLEGNRIKYLTNSTFLSCDSLTVLFLPRNQIGFVPEKTFSSLKNLGELDLSSNTITELSPHLFKDLKLLQKLNLSSNPLMYLHKNQFESLKQLQSLDL

**Supplementary Table S1.** Patient characteristics of the adrenal gland samples used in the RXFP2 transcript analysis in hypertensive men and normotensive men.

| **Specimen ID** | **Hypertension** | **Age** | **Sex** | **Ethnicity** | **BMI** | **Recovery type** |
| --- | --- | --- | --- | --- | --- | --- |
| 1153191F | Yes | 63 | Male | Caucasian | 26.81 | Autopsy |
| 1151587F | Yes | 74 | Male | Caucasian | 35.19 | Autopsy |
| 1138051F | Yes | 62 | Male | Caucasian | 26.4 | Autopsy |
| 1214368F | No | 28 | Male | Caucasian | 21.3 | Autopsy |
| 1205494F | No | 65 | Male | Caucasian | 38.89 | Autopsy |
| 1227699F | No | 68 | Male | Caucasian | 16.6 | Autopsy |
| 7949D1 | Yes | 80+ | Male | Caucasian | 22.2 | Autopsy |
| 8260C1 | Yes | 64 | Male | Caucasian | 27.5 | Autopsy |
| 1205516F | No | 65 | Male | Caucasian | 38.9 | Autopsy |
| 120879F | No | 28 | Male | Caucasian | 38.9 | Autopsy |
| 1210922F | No | 70 | Male | Caucasian | 17.9 | Autopsy |
| 1246653F | Yes | 55 | Male | Caucasian | 29.8 | Autopsy |

**Supplementary Table S2.** Amino acid sequences of RXFP2 mAbs.

| **mAb** | **Chain** | **Amino Acid Sequence** |
| --- | --- | --- |
| 1F2 | VH | QVQLKQSGPGLVQPSQSLSITCTVSGFSLTAYGVNWVRQSPGKGLEWLGVIWSGGNTDYNAPFISRLSITKDNSKSQVFFKMNSLEADDTAIYYCARAKGYYAMDYWGQGTSVTVSS |
|  | VL | DIQMTQSPASLSVSVGESVTITCRATENIYSNLVWYQQKQGQSPQLLVYAVTSLADGVPSRFSGSGSGTQYSLKINSLQSEDFGTYYCQHFWGTPYTFGGGTKLEIK |
| 1G1 | VH | EVQLQQSGPELVKPGASVKMSCKASGYTFTDYNMHWVKQKPGQGLEWIGYINPYNDGTEYNEKFKGKATLTSDKSSSTAYMDLSSLTSEDSAVYYCARGLWFAMNYWGQGTSVTVSS |
|  | VL | DIQMTQSSSSFSVSLGDRVTITCKASEDIYNRLAWYQQKPGNTPRLLISGATILETGVPSRFSGSGSGKDYTLSIISLQTEDVATYYCQQYWIIPPTFGGGTKLEIK |
| 3F3 | VH | QVQLQQPGSVLVRPGASVKLSCKASGYIFTSYWMHWVKQRPGQGLEWIGEIYPNSGRTSYNEKFKGKATVTVDTSSSTAYVDLSSLTSEDSAVYYCASGGHWFAYWGQGTLVTVSA |
|  | VL | DIQMTQSSSSFSVSLGDRVTITCKASEDIYNRLAWYQQKPGNAPRLLISGATSLEAGVPSRFSGSGSGKDYTLSITSLQTEDVSTYYCQQYWITPYTFGGGTKLEIK |
| 4A5 | VH | DVQLVESGGGLVQPGGSRKLSCAASGFTFSTFGMHWVRQAPEKGLEWVAYISSGSSTIYYADTVKGRFTISRDNPKNTLFLQMTSLRSDDTAMYYCSRSYYGSAHYFDYWGQGTTLTVSS |
|  | VL | DIQMTQSPASLSVSVGETVTITCRASENIYSNLAWYQQKQGKSPQLLVYVATNLADGVPSRFSGSGSGTQYSLRINNLQSEDFGNYYCQHFWGTPPTFGGGTKLEIK |
| 4B8 | VH | QVQLKQSGPGLVQPSQSLSITCTVSGFSLSRYGVHWVRQSPGKGLEWLGVIWSGGSTEYNAVFKSRLSISKDNSKSQVFFKMNSLQANDTAIYYCVRADGYYAMEYWGQGTSVTVSS |
|  | VL | DIQMTQSPASLSVSVGETVTITCRASENIYSNLAWYQQKQGKSPQLLVYAVTNLADGVPSRFSGSGSGTQYSLKINSLQSEDFGNYYCQHFWGTPYTFGGGTKLEIK |
| 2D4 | VH | QVQLKESGPGLVAPSQSLSITCTVSGFSLTTYGISWVRQPPGKGLEWLGVIWTGGGTNYNSALKSRLSISKDNSKSQVFLKMNSLQTDDTARYYCARTEGYYVMDYWGQGTSVTVSS |
|  | VL | DIQMTQSPASLSVSVGETVTITCRASENIYSNLVWYQQKQGKSPQLLVYAATNLADGVPSRFSGSGSGTQYSLKINNLQSEDFGTYYCQHFWGPPWTFGGGTKLEIK |
| 3F1 | VH | QVQLKESGPGLVAPSQSLSITCTVSGFSLTDYGVSWIRQPPGKGLEWLGVIWGGGSTDYNPALKSRLSISKDNSKSQVFLKMNSLQADDTAMYYCVKHEGYYAMDYWGQGTSVTVSS |
|  | VL | DIQMTQSPASLSVSVGETVTITCRASENIYSNLAWYQQKQGKSPHLLVYAVTNLADGVPSRFSGSGSGTQYSLKINSLQSEDFGSYYCQHFWGTPYTFGGGTKLEIK |
| 3G4 | VH | QVQLKESGPGLVAPSQSLSITCTVSGFSLTSYGVSWVRQPPGKGLEWLGVIWGDGSTNYHSALISRLSISKDNSKSQVFLKLNSLQTDDTATYYCAKSLGYYAMDYWGQGTSVTVSS |
|  | VL | DIQMTQSPASLSVSVGETVTITCRASENIYSNLVWYQQKQGKSPQLLVYAATNLAEGVPSRFSGSGSGTQYSLKINSLQSEDFGSYYCQHFWGPPWTFGGGTKLEIK |
| 3H1 | VH | QVQLKETGPGLVAPSQSLSITCTVSGFSLTNYGVNWVRQPPGKGLEWLVVIWSDGSTNYNSALKSRLSISKDNSKSQVFLKMNSLQTDDTAMYYCARQQGYYAMDYWGQGTSVIVSS |
|  | VL | DIQMTQSPASLSVSVGETVTITCRASENIYSNLAWYQQKQGKSPQLLVYAVTNLADGVPSRFSGSGSGTQYSLKINSLQSEDFGSYYCQHFWGPPYTFGGGTKLEIK |
| 4G7 | VH | EVKLVESGGGLVQPGASLRLSCATSGFTFTDYYMSWVRQPPGKALEWLGFIRNKATGYTTEYSASVKGRFTISRDNSQSILYLQMNTLRAEDSATYYCARDLYYGDWYFDVWGAGTTVTVSS |
|  | VL | DIVLTQSPASLAVSLGQRATISCRASESVDKYGLSFMNWFQQKPGQPPKLLIYAASNQGSGVPARFSGSGSGTNFSLNIHPMEEDDTAMYFCQQSKEVPRTFGGGTKLEIK |
| 4H2 | VH | QVQLRQSGPSLVQPSQSLSITCTVSGFSLTNYGVHWVRQSPGKGLEWLGVIWRGGNTDHNAAFMSRLSITKDNSESQVFFKMNSLQVDDTAIYYCAKAEGYYALDYWGQGTSVTVYS |
|  | VL | DIQMTQSPASLSVSVGETVTITCRASENIYSNLAWYQQKQGKSPQLLVYAVTNLADGVPSRFSGSGSGTQYSLKINSLQSEDFGSYYCQHFWGTPWTFGGGTKLEIK |
| 4H5 | VH | QVQLKQSGPGLVQPSQSLSITCTVSGFSLTNYGVHWVRQSPGKGLEWLGVIWRDGNTDYNAAFMSRLSITKDNSKSQVFFEMNSLQVDDTAIYYCCRGDGYYAMGYWGQGTSVTVSS |
|  | VL | DIQMTQSPASLSVSVGETVTITCRASENIYSNLAWYQQRQGRSPQLLVYAATNLADGVPSRFSGSGSGTQYSLKINSLQSEDFGNYYCQHFWGSPRTFGGGTKLEIK |
| 2H3 | VH | QVTLKESGPGILQPSQTLSLTCSFSGFSLSTSGMSVGWIRQPSGKGLEWLAHIWWNDDKYYNPALKSRLTISKDTSNNQVFLKIASVVTADTATYYCARIGYYDGSPHAMDYWGQGTSVTVSS |
|  | VL | DIVLTQSPASLAVSLGQRATISCRASESVDNYGFSFMNWFQQKPGQPPKLLIYAASNQGSGVPARFSGSGSGTDFSLNIHPMEEADTAMYFCQQSKEVPWTFGGGTKLELK |
| 4B1 | VH | QVQLQQSGAELVRPGSSVRISCKASGYAFSNSWINWVKQRPGQGLEWMGQIYPGDGNTNYNGRFKDKATLTADKSSSTAYMQLSSLTSEDSAVYFCARDHNYWGQGTTLTVSS |
|  | VL | DVVMTQTPLTLSVTIGQPASISCKSSQSLLYSNGKTYLNWLLQRPGQSPKRLIYLVSYLDSGVPDRFTGSGSGTNFILNISRVEADDLGVYYCLQGTHFPRTFGGGTKLEIK |
| 4F6 | VH | EVQLQQSGPELVKPGASVKMSCKASGYTFTNYVLHWVKQKPGQGLEWIGYINPYNDGSKYNEKFKGRATLTSDKSSSTAYMELSSLTSEDSAVYYCARGGWLLDYFDYWGQGTTLTVSS |
|  | VL | DVLMTQSPLSLPVSLGEQASISCRSSQSIVHSDGDTYLEWYLQKPGQSPNLLIYKVSNRFSGVPDRFSGSGSGTDFTLKISRVEAEDLGVYYCFQGSHVPPTFGGGTKLEIK |
| 4G6 | VH | EVQLQQSGPELVKPGASVKLSCKASGYTFTSYVMHWVKQKPEQGLEWIGYKNPSNDGIKYNEKFKGKATLTSDKSSNIAYMELFSLTSEDSAVYYCARSDFGSVWAMDYWGQGTSVTVSS |
|  | VL | SIVMTQTPKFLLVSAGDRVTITCKASQSVNDDIVWYQQKPGQSPKLLIYYASNRYTGVPDRFTGSGYGTDFTFTISTVQAGDLAVYFCQQDYSSPFTFGSGTKLEIK |
| 4H1 | VH | QIQLVQSGPELKKPGETVKISCKTSGYTFTKYPMHWVKQAPGEGLKWMGWINTYSGLPTFADDFKGRFAFSLETSANTADLQINNLKNEDMATYFCAREGKKLSGYFDYWGQGTTLTVSS |
|  | VL | DIKMTQSPSSMYASLGERVTITCKASQDINSYLTWFQQKPGQSPKTLIYRANRFVDGVPSRFSGSGSGQDYSLTITSLEYEDMGIYYCLQYDEFPYTFGGGTQLEIK |

**Supplementary Methods.** Methods used for characterization of HTS and ELT compounds in Figures S6-S11.

NMR spectra were recorded at ambient temperature unless otherwise stated using standard pulse methods on any of the following spectrometers and signal frequencies: Bruker V-400 or Bruker AV-400 (^1^H = 400 MHz). Chemical shifts are reported in ppm and are referenced to tetramethylsilane (TMS) or the following solvent peaks: CDCl_3_ (^1^H = 7.27 ppm), DMSO-*d*_6_ (^1^H = 2.50 ppm). Coupling constants are quoted to the nearest 0.1 Hz, and multiplicities are given by the following abbreviations and combinations thereof: s (singlet), d (doublet), t (triplet), q (quartet), m (multiplet), and br (broad). The spectra were analyzed by ACD/Spectrus Processor 2017.2.

LCMS analysis for compounds GSK618069 I3, GSK618069 I4, and BRL-37274 were carried out on a Waters Acquity UPLC instrument equipped with a Acquity UPLC CSH C18 (50 mm × 2.1 mm, 1.7 μm packing diameter) and Waters micromass ZQ MS using alternate-scan positive and negative electrospray. Analytes were detected as a summed UV wavelength of 210–350 nm. Two liquid phase methods were used: Formic: run at temperature 40°C, 1 mL/min flow rate. Gradient elution with the mobile phases as (A) water containing 0.1% volume/volume (v/v) formic acid and (B) acetonitrile containing 0.1% (v/v) formic acid. Gradient conditions were initially 3% B, increasing linearly to 97% B over 1.5 min, remaining at 97% B for 0.4 min, and then increasing to 98% B over 0.1 min. High pH: run at temperature 40°C, 1 mL/min flow rate. Gradient elution with the mobile phases as (A) 10 mM aqueous ammonium bicarbonate solution, adjusted to pH=10 with 0.88 M aqueous ammonia and (B) acetonitrile. Gradient conditions were initially 3% B, increasing linearly to 95% B over 1.5 min, remaining at 95% B for 0.4 min, and then increasing to 97% B over 0.1 min.

Analysis for compounds BRL-37274 I5 and I6 were carried out on an Agilent 1260 HPLC Column; Agilent Poroshell 120SB-C18, 3.0x30mm 2.7 μM. Temp 40°C, 0.04% TFA in water, 0.02% TFA in ACN Flow rate 2 mL/min. Gradient conditions were initially 5% B, increasing linearly to 95% B over 0.8 min, remaining at 95% B for 0.4 min, and then decreasing to 5% B over 0.01 min.

Analysis for compound BRL-37274 I4 was carried out on a Shimadzu LC-20AD MSD:LCMS-2020 Column Kinetex EVO C18, 2.1 x 30mm 5 μM. Temp 40°C, 0.04% TFA in water, 0.02% TFA in ACN Flow rate 1.5 mL/min. Gradient conditions were initially 5% B, increasing linearly to 95% B over 0.69 min, remaining at 95% B for 0.46 min, and then decreasing to 5% B over 0.34 min.

Analysis for compounds GSK4761528, GSK4720639, GSK4762707 and GSK4762706 were carried out on a Accela PDA (Scan range: 200-400 nm) Thermo LCQ Fleet (Positive ionization, scan range: 150-1600 m/z) **Column:**Kinetex C8; 2.1 x 30 mm; 1.7 μM; 100 Å **Mobile Phase A:** water w/ 0.1% formic acid m**obile phase B:** acetonitrile w/ 0.1% formic acid.

Purifications by normal phase column chromatography were performed on prepacked silica gel columns using biotage SP4, Isolera One, or Teledyne ISCO apparatus. Mass-directed automatic purification (MDAP) was carried out using a Waters ZQ MS using alternate-scan positive and negative electrospray and a summed UV wavelength of 210–350 nM. Solvents used for mobile phase will be described when used.

Reverse phase purification was performed by MDAP or HPLC. Formic MDAP Formic: Xselect CSH C18 column (150 mm x 30 mm i.d. 5 μm packing diameter) at ambient temperature, 40 mL/min flow rate). Gradient elution at ambient temperature with the mobile phases as (A) water containing 0.1% volume/volume (v/v) formic acid and (B) acetonitrile containing 0.1% (v/v) formic acid. Injection volume 1 mL (used for GSK618069, GSK4761528, GSK4720639, GSK4762707 and GSK4762706). Formic HPLC details: Instrument: Shimadzu LC-8A preparative HPLC. Column: Phenomenex Luna C18 75 mm x 30 mm x 3μm. Mobile phase: A for water (0.1% formic acid) and B for CAN. Flow rate: 25 mL/min. Wavelength: 220 & 254 nm. Gradient conditions were initially 30% B, increasing linearly to 70% B over 8 min, remaining at 70% B for 0.1 min, increasing to 100% B over 0.1 min holding at 100% B for 2 min then decreasing to 30 %B over 0.1 min and holding at 30% B for 1.2 min.  (used for BRL-37274 I6 and BRL-37274).
